# Retinal vasculometry associations with cognition status in UK Biobank

**DOI:** 10.1002/dad2.70087

**Published:** 2025-02-24

**Authors:** Royce Shakespeare, Alicja R. Rudnicka, Roshan Welikala, Sarah A. Barman, Anthony P. Khawaja, Paul J. Foster, Christopher G. Owen, the UK Biobank Eye and Vision Consortium

**Affiliations:** ^1^ Population Health Research Institute, St George's School of Health and Medical Sciences City St. George's, University of London London UK; ^2^ School of Computer Science and Mathematics Kingston University Kingston upon Thames Surrey UK; ^3^ NIHR Biomedical Research Centre Moorfields Eye Hospital NHS Foundation Trust and UCL Institute of Ophthalmology London UK

**Keywords:** cognition, neurovascular biomarker, retinal vasculometry

## Abstract

**Introduction:**

Retinal vasculometry (RV) provides a neurovascular biomarker which may relate to cognitive status. However, the presence and form of association remains unclear and unexamined at scale.

**Methods:**

Artificial intelligence–enabled RV measures from 66,350 UK Biobank study participants were related to combined cognition scores. Differences in RV were examined per standard deviation (SD) increase in cognitive score, using multilevel linear regression, adjusted for age, sex, measurement center, ethnicity, and within‐person RV clustering.

**Results:**

One hundred ten thousand two hundred eighty‐two retinal images from 63,165 (95%) participants (mean age 56.6 years, 55.5% female) were analyzed. A one SD increase in cognition score was strongly associated with increased arteriolar width, arteriolar tortuosity, increased venular width particularly among those < 50 years and venular area among those > 50 years; also, inversely associated with venular tortuosity, and arteriolar and venular width variance.

**Discussion:**

These easily accessible, affordable, and non‐invasive RV measures should be evaluated further as an early predictor of future neurodegenerative disease.

**Highlights:**

- How cognitive status relates to retinal vasculometry (RV) measures remains uncertain and unexamined at scale.
- Using data from a large population‐based study (UK Biobank) we show strong graded associations between cognitive status and RV, which contrast with some RV associations observed with aging. Specifically, increased arteriolar tortuosity, arteriolar and venular width (at younger ages), and area are positively associated, and venular tortuosity and arteriolar and venular width variability are inversely associated with higher cognitive status, all showing strong, graded, precise relationships. These associations appeared to be strongest for fluid intelligence and prospective memory tests.
- These easily accessible, non‐invasive RV measures provide a neurovascular marker indicative of cognitive status, which should be evaluated as early predictors of neurodegenerative disease.

## 1 BACKGROUND

Alzheimer's disease (AD), the most common form of dementia, is a neurodegenerative disorder that is a major cause of death and disability (leading to loss of functional independence) among older people worldwide.^1^ Disease numbers are increasing with an ever‐aging population, often as part of a growing burden of multi‐morbidities.^2^ Early detection by recognizing cognitive decline may well be key in preventing this trend,^1^ especially as the majority of candidate drugs for slowing cognitive decline in AD or other dementias have failed in clinical trials, probably because they are used too late in the disease process.^3^ Hence, early warning systems for neurodegenerative disease are needed to identify those at high risk, to intervene early.^3^ In terms of the microcirculatory system, an association between microvascular disease and AD is increasingly recognized.^4^, ^5^ *Post mortem* studies of the cerebral microvasculature in persons with AD have shown impairment of the blood–brain barrier and decreased capillary density, length, and mean diameters compared with controls.^6^, ^7^ Cerebral microvascular changes are currently only accessible to in vivo imaging technologies by magnetic resonance imaging and positron emission tomography (PET). However, the retinal vasculature is a direct extension of the intracranial circulation, providing a more readily accessible neurovascular marker.^8^, ^9^, ^10^, ^11^, ^12^, ^13^ This has led to previous attempts to discover associations between AD and various vascular parameters in color fundus photographs (CFP).^14^ However, findings have been limited and equivocal to date,^15^ and to the best of our knowledge have not been examined at scale. Previous work has shown associations between eye disease and AD,^16^ and shown retinal arterial and venule occlusion associations with vascular dementia.^17^ Other studies at a capillary level, based on optical coherence tomography angiography (OCT‐A) have more recently emerged.^18^ However, while findings from these studies using OCT‐A suggest decreased vascular density associated with AD, findings from different studies have been inconsistent,^18^, ^19^, ^20^ and longitudinal studies are needed to demonstrate cause and effect.^19^, ^20^ Moreover, the link between OCT‐A and cognitive status as a precursor of neurodegenerative disease remains to be established.

Advances in retinal image analysis, particularly harnessing developments in artificial intelligence (AI)–based approaches, have afforded automated extraction of detailed retinal vasculometry (RV) characteristics, allowing application to large population‐based studies.^21^, ^22^, ^23^ This technology has not only allowed associations between RV phenotypes and disease risk and outcomes to be examined definitively at scale,^24^, ^25^, ^26^ but has also allowed the role of RV particularly in circulatory and vascular disease prediction to be realized.^27^ In addition to retinal feature detection approaches, end‐to‐end AI approaches have also been used in risk factor and disease detection,^28^, ^29^ with application to retinal disease.^30^ While end‐to‐end AI approaches have recently been used to detect AD from CFPs among those with established disease (compared with those without disease),^31^ the use of such approaches to predict disease (i.e., in early “prodromal” stages) is yet to be established, especially in large population settings. A recent study has shown promise in discerning participants with mild cognitive impairment compared with participants without, but only from OCT and OCT‐A images.^32^ Hence, we examined RV associations with cognitive status, as an early marker of neurodegenerative disease, in a large well‐characterized nationally representative population.

## 2 METHODS

UK Biobank is a large prospective cohort, designed to improve the prevention, diagnosis, and treatment of disease. Baseline assessments were carried out 2006 through 2010, in 502,682 adults aged 40 to 69 years recruited from 22 UK centers, including questionnaire, physical measurements, and collection of biological samples.^33^ Details of the physical examination, blood measures, and collection of other data (including health outcome data) have been detailed elsewhere.^33^

**Research in context**

1. **Systematic review**: A systematic review of the literature (using PubMed) was used to identify all relevant literature relating retinal vessel size and shape, so called “retinal vasculometry” (RV), to cognitive status and neurodegenerative outcomes. We have cited findings to date, but these have been limited and equivocal, and to the best of our knowledge have not been examined at scale.
2. **Interpretation**: Our findings add to the literature showing strong graded associations between automated artificial intelligence measures of RV and a combined cognitive status score. These associations were independent of the strong associations observed with age, and were particularly evident for fluid intelligence and prospective memory components of the combined score, suggestive of regional neuro‐specific pathways which might underpin the association.
3. **Future directions**: Given the low cost, and rapid, non‐invasive, and readily accessible availability of retinal imaging (particularly within existing opticians and eye clinic health‐care pathways), this technology should be evaluated further as a neurovascular biomarker predictive of neurodegenerative outcome, to maximize opportunities for early treatment that could avert/delay progression to neurodegenerative disease.

### 2.1 Cognitive status

Cognitive testing was included in the baseline assessment as part of a fully automated touchscreen questionnaire.^33^ Assessment included a wide range of cognitive function tests that are relevant for assessing various aspects of cognitive decline and dementia phenotype (including fluid IQ, pairs matching, prospective memory, reaction time, numeric memory, matrix, symbol digit substitution, tower test, trail making, paired associate learning).^10^, ^34^ The reliability and validity of these non‐standard cognitive tests, developed specifically for UK Biobank, have been subsequently shown.^34^, ^35^ Using principal component analysis (PCA), these different cognitive measures can be combined to provide a general measure of cognitive ability, which has been shown to correlate highly with established cognitive measures.^34^, ^35^ The same method was followed to derive a general measure of cognitive ability among those who underwent ocular examination. Among those who underwent ocular assessment, scores from pairs matching (log [x+1] transformed), reaction time (log transformed), prospective memory, and fluid intelligence tests were identified from PCA and used to derive a composite cognitive score. Only participants with all four tests completed and retinal images taken for one or both eyes at their respective visits were included in the PCA. Description of the tests used to derive a general cognitive score (G4) are provided in Table S1 in supporting information. The derived score, the first unrotated component of the PCA, had an eigenvalue of 1.53, which accounted for 38% of the variance (Table S2 in supporting information, scree plot in Figure S1 in supporting information). The factor loadings of the individual tests were > 0.4 for all tests (Table S3 in supporting information). The PCA‐derived G4 score showed a slight negative skew (Figure S2 in supporting information). The general cognitive score was named G4 to indicate that it was derived from four cognitive tests available in the UK Biobank.

### 2.2 Ocular examination

Ocular assessments were carried out from 2009.^36^ Digital CFPs were captured using the Topcon 3D‐OCT 1000 Mark 2. A single non‐mydriatic, 45° digital color image, centered on the fovea, was captured at the baseline visit for 68,550 participants (135,867 images from one or both eyes).^36^ Further images were obtained from 17,534 participants who attended for reassessment, 1 to 5 years after the baseline assessment (late 2012 to mid‐2013). Overlap with baseline ocular assessment was minimal as recruitment centers revisited did not include those that had ocular image capture at baseline.

### 2.3 AI‐enabled RV assessment

Image processing was carried out using a fully automated computerized system (QUARTZ [Quantitative Analysis of Retinal vessel Topology and size], Figure S3 in supporting information). QUARTZ distinguishes between right and left eyes (by optic disc localization) and between venules and arterioles, identifies vessel segments, outputs centerline coordinates, and provides thousands of measures of vessel width (µm), area (mm^2^), precision (1/[standard deviation (SD) of within‐vessel widths]) and tortuosity (arbitrary units). Image level averages weighted by vessel segment length were used. QUARTZ has been extensively used and validated, having thus far measured 11 million vessel segments from > 200,000 images from 100,000 participants.^24^, ^25^, ^26^, ^27^, ^37^ A model eye was used to quantify the magnification characteristics of the telecentric fundus camera used (Topcon 3D‐OCT 1000 Mark 2), allowing pixel dimensions of vessel width to be converted to real size.^38^

### 2.4 Statistical analysis

Retinal arteriolar and venular diameters were normally distributed; tortuosity required log transformation. Multilevel linear regression models adjusted for age, sex, ethnicity, and UK Biobank center as fixed effects, with a random effect for person to allow for repeated RV measures within the same person (model 1), were used to examine associations of combined cognitive score with vasculometry outcomes. Model 2 additionally adjusted for smoking status, Townsend Deprivation Index, and height (cm); height (which relates to brain size and cognition)^39^ was fitted as a further marker of social status. Model 3 included further adjustment for body mass index (BMI; kg/m^2^), hemoglobin A1c (HbA1c; mmol/mol), systolic blood pressure (mmHg), total cholesterol (mmol/L), and triacylglycerol (mmol/L). Model 4 examined the exclusion of those with self‐reported history of myocardial infarction, stroke, hypertension, or on medication for hypertension. Levels of adjustment followed the approach we have used previously,^37^ allowing for non‐modifiable risk factors, followed by socioeconomic‐related factors, then cardiometabolic risk factors, examining the effect of exclusion of cardiovascular outcomes. Models identical to those fitted above with further adjustment for educational qualification and refractive error were examined but made little difference. RV associations with individual test scores used to generate G4 (including fluid intelligence and prospective memory tests) were carried out as complementary analysis. Data missing on categorical variables were included as an additional category for each variable, to minimize data loss. Associations with the log‐transformed tortuosity were exponentiated to give percentage differences in vessel tortuosity, and absolute differences in vessel width, area, and precision per specified increase in exposure variable (per SD for G4). Interactions between cognitive score and RV measures were examined for sex, age, and ethnic group. Given the large sample size of UK Biobank *P* values for statistically significant interactions were set to < 0.001 (which given 32 tests for interaction approximates to a Bonferroni correction). Statistical analyses were carried out using STATA (version 17, StataCorp LP).

## 3 RESULTS

Of the 88,052 UK Biobank participants who underwent retinal imaging, 66,350 passed QUARTZ image quality assessment (75%), of which 63,195 (72%) with cognitive scores (110,282 images) were included in the analysis (Figure S4 in supporting information). Mean age was 56.5 years, 55.1% were female, and most (93.2%) were of White ethnic origin (Table 1). Compared with those excluded, participants included with complete data were slightly younger and more likely to be women; less likely to be a current smoker; less deprived; had lower BMI, HbA1c, and systolic blood pressure; and were less likely to be hypertensive (Table 1). Those included were also less likely to have had a heart attack or stroke (Table 1). RV characteristics were broadly similar, although those included had thinner retinal arterioles and wider venules (Table 1). Those included were also taller compared with those excluded (Table 1).

**TABLE 1:** UK Biobank population characteristics overall and among those included and excluded from analyses.

Characteristics	Total	Included	Excluded
**N**	66350	63165	3185
**Age (SD) years**	56.6 (8.2)	56.5 (8.2)	57.4 (8.6)
**Sex, *N* (%) female**	36489 (55.0)	34788 (55.1)	1701 (53.4)
**Ethnicity, *N* (%)**			
White	61036 (92.0)	58860 (93.2)	2176 (68.3)
Black	1640 (2.5)	1364 (2.2)	276 (8.7)
Asian	1646 (2.5)	1329 (2.1)	317 (10.0)
Other	1638 (2.5)	1398 (2.2)	240 (7.5)
Unknown/didn't answer/missing	390 (0.6)	214 (0.3)	176 (5.5)
**Smoking, *N* (%)**			
Never	37592 (56.7)	35822 (56.7)	1770 (55.6)
Occasionally	1749 (2.6)	1653 (2.6)	96 (3.0)
Previous	22595 (34.1)	21723 (34.4)	872 (27.4)
Current	4052 (6.1)	3789 (6.0)	263 (8.3)
Prefer not to answer or missing	362 (0.5)	178 (0.3)	184 (5.8)
**Quartiles of Townsend Deprivation Index, *N* (%)**			
<−3.4	16605 (25.0)	16082 (25.5)	523 (16.4)
−3.4 to −1.6	16585 (25.0)	15948 (25.2)	637 (20.0)
−1.7–0.8	16495 (24.9)	15780 (25.0)	715 (22.4)
> 0.8	16587 (25.0)	15282 (24.2)	1305 (41.0)
Missing	78 (0.1)	73 (0.1)	5 (0.2)
**BMI (kg/m^2^)**	27.2 (4.7)	27.1 (4.7)	27.7 (4.8)
**Arteriolar width (µm)**	86.9 (8.5)	86.8 (8.5)	87.1 (8.6)
**Venular width (µm)**	103.0 (14.1)	103.0 (14.0)	101.4 (15.5)
**Arteriolar area (mm^2^)**	1.8 (0.9)	1.9 (0.9)	1.8 (0.8)
**Venular vessel area (mm^2^)**	2.5 (0.9)	2.5 (0.9)	2.4 (1.0)
**Precision arteriolar diameter SD^−1^**	0.1 (0.03)	0.1 (0.03)	0.1 (0.03)
**Precision venular diameter SD^−1^**	0.1 (0.03)	0.1 (0.03)	0.1 (0.03)
**Arteriolar tortuosity** ^a^	1.5 (0.5)	1.5 (0.5)	1.4 (0.5)
**Venular tortuosity** ^a^	1.1 (0.4)	1.1 (0.3)	1.2 (0.4)
**Height (cm)**	168.7 (9.2)	168.8 (9.2)	166.2 (9.1)
**HbA1c (mmol/mol)**	35.9 (6.3)	35.8 (6.1)	37.6 (8.3)
**Systolic blood pressure (mmHg)**	136.6 (18.3)	136.4 (18.2)	139.4 (19.6)
**Cholesterol (mmol/L)**	5.7 (1.1)	5.7 (1.1)	5.5 (1.1)
**Triglycerides (mmol/L)**	1.7 (1.0)	1.7 (1.0)	1.7 (1.0)
**Prospective memory test *N*, (%)**	52185 (78.7)	52185 (82.6)	–
**Pairs matching (no. of errors)**	4.0 (3.2)	4.0 (3.2)	–
**Reaction time (ms)**	559.5 (117.2)	559.5 (117.2)	–
**Fluid final (no. of questions answered correctly)**	6.2 (2.2)	6.2 (2.2)	–
**G4 (arbitrary units)**	0.0 (1.2)	0.0 (1.2)	–
**Stroke, *N* (%)**	914 (1.4)	814 (1.3)	100 (3.1)
**Heart attack, *N* (%)**	1261 (1.9)	1165 (1.8)	96 (3.0)
**High blood pressure and medication, *N* (%)**	18021 (27.2)	16962 (26.9)	1059 (33.3)

*Note*: Mean (SD) unless otherwise stated.

Abbreviations: BMI, body mass index; G4, general cognitive score; SD, standard deviation.

^a^ Natural log‐transformed values.

**FIGURE 1:**
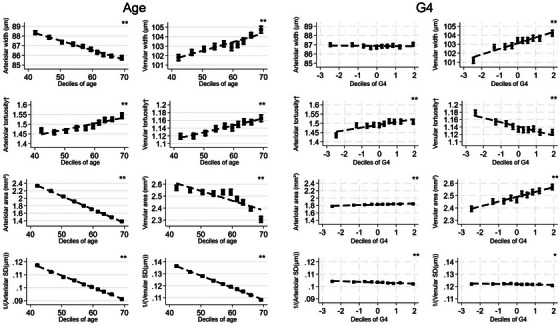
Adjusted mean RV characteristics by deciles of age (left) and G4 (right). Adjusted means (solid square symbols), 95% CIs (solid vertical error bars), and regression line (dashed line) for RV, by deciles of age (left) and G4 (right), from multilevel models adjusted for age and sex, allowing for repeated RV measures within each person (*N* participant = 63,165, *n* images = 110,282). † denotes natural log‐transformed values. * denotes statistical significance for a trend test across deciles of age/G4 (0.001 ≤ *P* value < 0.05) ** denotes statistical significance for a trend test across deciles of age/G4 (*P* value < 0.001). CI, confidence interval; G4, general cognitive score; RV, retinal vasculometry

Figure 1 shows adjusted mean RV characteristics by deciles of age and G4 score. Age showed strong graded inverse associations with arteriolar width, area, and precision, while it was positively associated with arteriolar tortuosity. For venular measures, age was inversely associated with venular area and precision but positively associated with venular width and tortuosity. G4 was positively associated with venular width and area, but inversely associated with venular precision and tortuosity. Additionally, G4 showed a positive association with arteriolar tortuosity and an inverse association with arteriolar precision. There was a modest positive association of G4 with arteriolar width, which persisted with adjustment (Table 2), whereas arteriolar width inverse associations with age were attenuated after adjustment (Table 2, models 3 and 4).

**TABLE 2:** Difference in RV characteristics per SD increase in general cognitive score (G4) and per decade in age, from multivariable regression models with different levels of adjustment.

	Model 1		Model 2		Model 3		Model 4	
	Difference (95% CI)	*P* value	Difference (95% CI)	*P* value	Difference (95% CI)	*P* value	Difference (95% CI)	*P* value
**Arteriolar width**								
per SD in G4	**0.16 (0.09,0.22)**	**1.49E^−06^**	**0.09 (0.03,0.15)**	**0.01**	**0.09 (0.02,0.16)**	**0.01**	**0.10 (0.02,0.18)**	**0.01**
per decade in age	**−0.81 (−0.89,−0.74)**	**4.44E^−96^**	**−0.61 (−0.69,−0.53)**	**3.58E^−52^**	−0.06 (−0.15,0.03)	0.19	−0.02 (−0.13,0.08)	0.66
**Venular width**								
***Age < 50***								
per SD in G4	**0.65 (0.43,0.87)**	**6.26E^−09^**	**0.65 (0.42,0.87)**	**1.55E^−08^**	**0.65 (0.41,0.90)**	**1.87E^−07^**	**0.59 (0.32,0.85)**	**1.34E^−05^**
per decade in age	0.58 (−0.21,1.36)	0.15	0.63 (−0.16,1.42)	0.12	**0.92 (0.06,1.78)**	**0.04**	0.73 (−0.19,1.64)	0.12
***50 ≤ Age < 60***								
per SD in G4	**0.55 (0.36,0.74)**	**1.33E^−08^**	**0.58 (0.39,0.77)**	**3.62E^−09^**	**0.57 (0.35,0.78)**	**1.84E^−07^**	**0.48 (0.23,0.73)**	**1.45E^−04^**
per decade in age	0.58 (−0.02,1.18)	0.06	**0.76 (0.15,1.37)**	**0.01**	**0.90 (0.22,1.58)**	**0.01**	**1.19 (0.40,1.97)**	**3.03E^−03^**
***Age ≥ 60***								
per SD in G4	0.09 (−0.07,0.25)	0.26	0.11 (−0.05,0.27)	0.17	0.11 (−0.06,0.29)	0.21	0.09 (−0.13,0.31)	0.42
per decade in age	1.72 (1.23,2.21)	**4.86E^−12^**	**1.87 (1.38,2.36)**	**7.50E^−14^**	**2.15 (1.59,2.71)**	**4.63E^−14^**	**2.48 (1.76,3.19)**	**9.50E^−12^**
**Arteriolar tortuosity** ^b^								
per SD in G4	**0.48 (0.06,0.89)**	**0.03**	**0.56 (0.14,0.98)**	**0.01**	**0.67 (0.20,1.14)**	**0.01**	**0.83 (0.27,1.39)**	**3.48E^−03^**
per decade in age	**2.25 (1.74,2.77)**	**2.59E^−18^**	**2.31 (1.79,2.84)**	**4.04E^−18^**	**1.18 (0.56,1.80)**	**1.90E^−04^**	**0.89 (0.16,1.61)**	**0.02**
**Venular tortuosity** ^b^								
per SD in G4	**−0.68 (−0.93,−0.44)**	**6.92E^−08^**	**−0.59 (−0.84,−0.34)**	**4.65E^−06^**	**−0.39 (−0.67,−0.11)**	**0.01**	−0.31 (−0.63,0.02)	0.06
per decade in age	**2.27 (1.96,2.58)**	**1.21E^−48^**	**2.37 (2.05,2.68)**	**1.86E^−49^**	**1.92 (1.55,2.30)**	**1.90E^−24^**	**1.78 (1.35,2.21)**	**4.14E^−16^**
**Arteriolar area**								
per SD in G4	**0.01 (0.00,0.02)**	**2.01E^−03^**	**0.01 (0.00,0.01)**	**0.04**	0.01 (0.00,0.01)	0.16	0.01 (0.00,0.01)	0.22
per decade in age	**−0.36 (−0.37,−0.35)**	**<10^−323^**	**−0.35 (−0.36,−0.35)**	**<10^−323^**	**−0.31 (−0.32,−0.31)**	**<10^−323^**	**−0.32 (−0.33,−0.31)**	**<10^−323^**
**Venular area**								
***Age < 50***								
per SD in G4	**0.02 (0.00,0.03)**	**0.03**	0.01 (0.00,0.03)	0.17	0.01 (−0.01,0.03)	0.32	0.01 (−0.01,0.03)	0.19
per decade in age	−0.03 (−0.08,0.03)	0.35	−0.01 (−0.07,0.04)	0.67	−0.01 (−0.07,0.05)	0.75	−0.03 (−0.09,0.04)	0.44
***50≤ Age < 60***								
per SD in G4	**0.05 (0.04,0.06)**	**8.75E^−14^**	**0.04 (0.03,0.05)**	**2.28E^−10^**	**0.05 (0.03,0.06)**	**1.77E^−10^**	**0.05 (0.04,0.07)**	**8.88E^−10^**
per decade in age	−0.16 (−4.21,3.88)^*^	0.94	0.01 (−0.03,0.05)	0.64	0.03 (−0.02,0.07)	0.23	−0.01 (−0.06,0.05)	0.80
***Age ≥ 60***								
per SD in G4	**0.04 (0.03,0.05)**	**6.60E^−14^**	**0.03 (0.02,0.04)**	**7.32E^−11^**	**0.03 (0.02,0.04)**	**4.38E^−09^**	**0.04 (0.02,0.05)**	**8.11E^−08^**
per decade in age	**−0.17 (−0.20,−0.14)**	**1.08E^−26^**	**−0.15 (−0.18,−0.12)**	**6.55E^−22^**	**−0.14 (−0.17,−0.10)**	**2.10E^−14^**	**−0.13 (−0.17,−0.08)**	**3.46E^−08^**
**1/ (arteriolar SD[µm])** ^a^								
per SD in G4	**−0.06 (−0.08,−0.04)**	**5.48E^−08^**	**−0.06 (−0.08,−0.04)**	**5.66E^−08^**	**−0.06 (−0.08,−0.04)**	**1.82E^−07^**	**−0.06 (−0.09,−0.03)**	**1.94E^−05^**
per decade in age	**−0.93 (−0.95,−0.90)**	**<10^−323^**	**−0.93 (−0.96,−0.91)**	**<10^−323^**	**−0.85 (−0.88,−0.82)**	**<10^−323^**	**−0.87 (−0.91,−0.84)**	**<10^−323^**
**1/ (venular SD[µm])** ^a^								
per SD in G4	**−0.05 (−0.08,−0.03)**	**6.04E^−06^**	**−0.05 (−0.08,−0.03)**	**5.53E^−06^**	**−0.05 (−0.08,−0.03)**	**7.15E^−05^**	**−0.05 (−0.08,−0.02)**	**1.00E^−03^**
per decade in age	**−1.03 (−1.06,−1.00)**	**<10^−323^**	**−1.04 (−1.07,−1.02)**	**<10^−323^**	**−1.04 (−1.07,−1.00)**	**<10^−323^**	**−1.04 (−1.08,−1.00)**	**<10^−323^**

*Note*: Model 1: adjusted for age, sex, ethnicity, and UK Biobank center. Model 2: Model 1 adjustment, with adjustment for smoking, Townsend Deprivation Index, and height. Model 3: Model 2 adjustment, with adjustment for BMI, HbA1c, systolic BP, total cholesterol, and triacylglycerols. Model 4:Same as Model 3 but excluding persons with self‐reported history of heart attack, stroke, hypertension, or on medication for hypertension. Results for venular width and venular area are stratified by age due to statistically significant interaction term (*P* < 0.001) between age and G4.

Abbreviations: BMI, body mass index; BP, blood pressure; CI, confidence interval; G4, general cognitive score; HbA1c, hemoglobin A1c; RV, retinal vasculometry; SD, standard deviation.

Bold text denotes statistically significant associations.

^a^ Coefficients multiplied by 100.

^b^ Coefficients as % change for log‐transformed target variables.

Arteriolar tortuosity associations with G4 were more marked after adjustment and exclusion of those with prevalent cardiovascular disease (1%/SD rise in G4), but associations for venular tortuosity were attenuated after adjustment (Table 2, models 3 and 4). Reduced arteriolar area with age (≈ 0.3 mm^2^/decade) was evident across all models.

Tests for interaction were statistically significant (*P* < 1 × 10^−8^ in all instances) between G4 and age with venular width and venular area, hence coefficients in Table 2 were stratified by age group for these associations.

**FIGURE 2:**
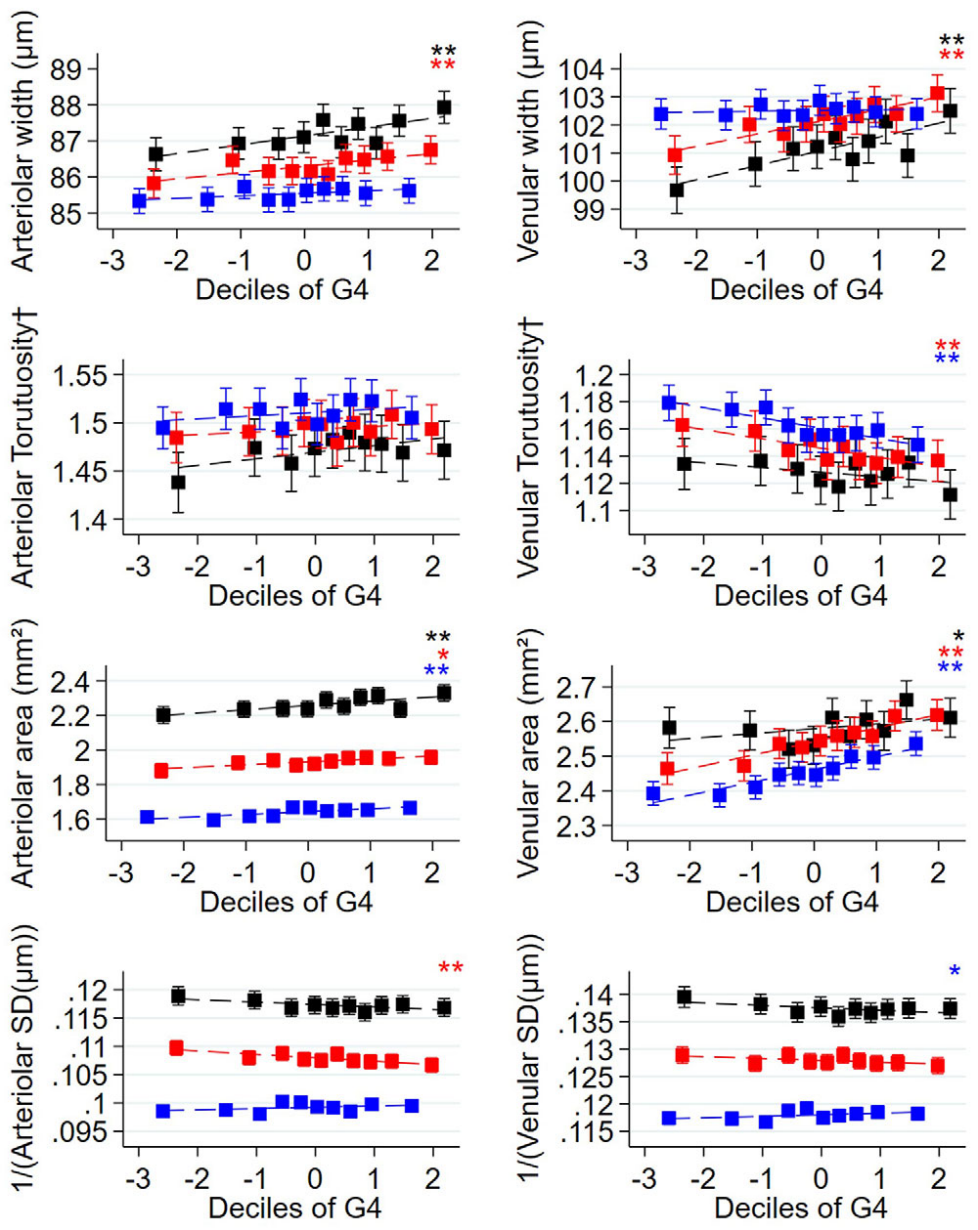
Adjusted mean RV characteristics by deciles of cognition scores by age group. Adjusted means (solid square symbols), 95% CIs (solid vertical error bars), and regression line (dotted line) for RV by deciles of G4 are from a multilevel model stratified by age category adjusted for sex, ethnicity, UK Biobank center as fixed effects, allowing for repeated RV measures within each person (*N* participants = 63,165, *n* images = 110,282). Black, red, and blue represent ages < 50, 50 to < 60, ≥ 60 years, respectively. † denotes natural log‐transformed values. * denotes statistical significance for a trend test across deciles of age/G4 (0.001 ≤ *P* value < 0.05) ** denotes statistical significance for a trend test across deciles of age/G4 (*P* value < 0.001). CI, confidence interval; G4, general cognitive score; RV, retinal vasculometry; SD, standard deviation

Venular width showed a consistent rise (≈ 0.5 µm) per SD rise in G4 across all models in the under 60 years age groups (Table 2 and Figure 2) whereas venular widening per decade in age was particularly strong in the oldest age group (≈ 2.0–2.5 µm/decade). Increased venular area with G4 was most marked in the 50+ age groups (≈ 0.05 mm^2^ per SD) and the venular area reduction of 0.2 mm^2^ per decade in age remained in the oldest age group after adjustment.

Venular tortuosity was consistently more strongly related to age (≈ 2 %/decade) compared with arteriolar tortuosity (≈ 1 %/decade) in adjusted analyses.

Both measures of vessel precision showed a consistent decline with rise in G4 particularly with rising age. Because of the inverse transformation, a decline in precision equates to increased variation in vessel widths along a vessel with rising age and G4.

For completeness, Figure 2 shows associations stratified by age groups for all RV measures and for all factors showing systematic differences between age groups, but that the patterns are broadly similar (except for venular width and area).

Full outputs from the regression models showing the independent contribution of other relevant covariates to RV characteristics (in particular, sex, height, HbA1c, and systolic blood pressure) are shown in Table S4 in supporting information. Further adjustment for educational status showed similar associations between G4 and RV (Table S5 in supporting information).

**FIGURE 3:**
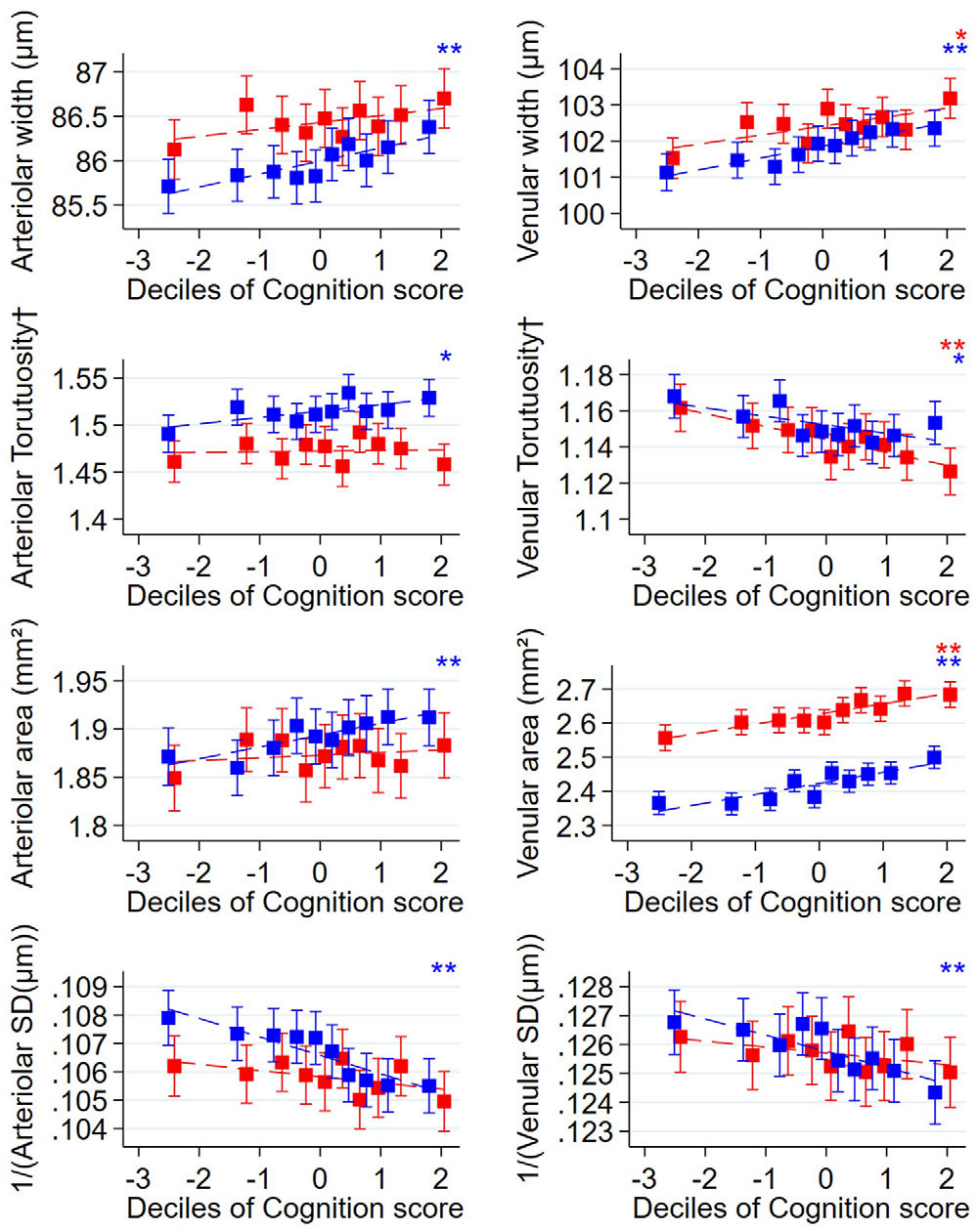
Adjusted mean RV characteristics by deciles of cognition scores by sex (red: male, blue: female). Adjusted means (solid square symbols), 95% CIs (solid vertical error bars), and regression line (dotted line) for RV by deciles of G4 are from a multilevel model stratified by sex adjusted for age, ethnicity, UK Biobank center as fixed effects, allowing for repeated RV measures within each person (*N* participants = 63,165, *n* images = 110,282). Red represents males while blue represents females. † denotes natural log‐transformed values. * denotes statistical significance for a trend test across deciles of age/G4 (0.001 ≤ *P* value < 0.05) ** denotes statistical significance for a trend test across deciles of age/G4 (*P* value < 0.001). CI, confidence interval; G4, general cognitive score; RV, retinal vasculometry; SD, standard deviation

Directions of G4 associations with RV showed similar patterns for men and women (Figure 3) with no formal evidence of interactions. However, men had wider arteriolar and venular width, and larger venular area compared with women. Levels of arteriolar tortuosity and arteriolar precision were higher in women, but there was no clear difference by sex in levels of association with venular tortuosity and venular precision (Figure 3).

Although proportionately UK Biobank includes participants predominantly of White ethnic background, the cohort still includes appreciable numbers from non‐White ethnic groups. RV characteristics by deciles of G4 among White participants, and by quintiles of G4 among those of Black and Asian ethnic origin are shown in Figure S5 in supporting information. Marked differences in absolute levels of RV by ethnic group were apparent, but directions of association of RV with G4 were broadly similar. Differences in RV characteristics per SD increase in G4 by ethnic group are shown in Table S6 in supporting information, and were broadly similar for White, Black, and Asian participants, with no formal evidence of a difference in association across ethnic groups.

For the individual tests used to derive the combined cognitive score, associations between RV and fluid intelligence score as well as prospective memory were similar to those found between RV and G4 (Table S7, Figure S6, and Table S8 in supporting information, respectively). However, no significant associations (*P* < 0.01) were identified between RV and both pairs matching and reaction time (Table S9, Figure S7, and Table S10, Figure S8 in supporting information, respectively).

## 4 DISCUSSION

While RV studies among those with AD (compared with those without) suggest a reduced vascular network associated with AD,^14^ and thinner arterioles and reduced vessel branching in wide‐field retinal images,^40^ associations with arteriolar and venular tortuosity remain unclear,^14^ and fewer studies have examined RV associations with cognitive decline.^14^ Among studies that have reported longitudinal findings, declines in cognitive status have been associated with reduced arteriolar^31^ and wider venular width,^31^, ^41^ but not consistently.^42^, ^43^ A concern is that many studies have lacked sufficient size to identify RV associations (if relationships truly exist), let alone identify the shape and form of any association. To the best of our knowledge, our findings are the first to report RV associations with cognitive status on this scale. While we showed no clear pattern of association between cognitive score and arteriolar width, we showed a clear graded positive association with venular width, among other definitive associations, which have not been previously described. We showed that lower levels of cognition, indicative of cognitive decline, were associated with reduced venular width, arteriolar and venular area, increased venular tortuosity, and arterial and venular precision. While these findings are commensurate with previous findings of a reduced vascular network associated with AD, other findings remain less clear, such as with precision, which opposes the association observed with aging. However, the totality of RV findings provide evidence of a link between microvascular changes and cognitive decline. Persistence of RV–cognitive score associations, particularly with fluid intelligence and prospective memory score components, may suggest regional as well as more general neuroanatomical correlates, although further vasculometry–brain volume analyses are needed.^44^, ^45^, ^46^ Moreover, G4 may not represent several domain‐specific cognitive domains (such as language or visuospatial processing) which may also contribute to lower performance on these tasks. Importantly the detailed extraction of vessel maps using QUARTZ allows more complex vasculometry characteristics to be extracted. This could provide a more in‐depth characterization of vessel complexity on an unprecedented scale, which could relate differently or more strongly to cognitive and ultimately neurogenerative status.^47^

The concept that the eye provides a window on neurodegenerative processes within the central nervous system is well established. Degeneration of the optic nerve is a well‐recognized feature of AD,^12^ and a growing body of evidence (including work carried out in UK Biobank) has shown that total and segmented thicknesses of the neuroretina give insights into both cognitive function and volumetric characteristics of the brain relevant to cognition.^8^, ^10^ People with AD have a thinner retinal nerve fiber layer (RNFL) and retinal ganglion cell layer (GCL) than those without AD of a similar age, and OCT measurement of RNFL and GCL may be useful in discriminating between AD and healthy individuals.^48^, ^49^ These findings provide a potential biological basis for the RV associations observed in that these neuronal changes may be as a result of or a consequence of reduced axonal activity and function.^13^ Moreover, the concept that the retina may allow early detection of systemic and non‐ocular disease (in addition to ocular neurodegenerative disorders, such as glaucoma) is well established for other vascular‐related diseases (see supporting information).^50^ We believe the combination of our unique method of RV assessment (as a marker of retinal vasculopathy and microvascular dysfunction) along with the strong graded associations observed, has a strong basis as a predictor of cognitive decline and subsequent neurodegenerative disease. Prediction approaches may need to include different RV characteristics and be nuanced to account for different vasculometry associations, for example, for venular width and venular area where there was evidence of effect modification in association by age. We have previously demonstrated such prediction approaches for circulatory mortality, coronary heart disease, and stroke in UK Biobank, which perform as well as established risk scores.^27^ For neurodegenerative disease, the additive value of RNFL along with retinal sub‐layer measures, may be incremental (see supporting information).

This study has strengths and weaknesses. UK Biobank offers one of the largest and most widely phenotyped retinal imaging data‐sets in the world.^36^ Even though macular centred retinal images were captured by non‐experts, inclusion rates were high, limiting potential selection biases.^27^ We also distinguished between arterioles and venules, which could show different RV–cognitive associations. While UK Biobank includes those largely of White ethnic origin, the numbers of participants of non‐White ancestry were still high, allowing the consistency of RV–cognitive associations across ethnic groups to be examined. However, replication of these findings in other large diverse data sources would still be worthwhile. UK Biobank is a “healthy” cohort compared with other similarly aged nationally representative cohorts (see supporting information), and appreciable numbers with AD are yet to evolve, although the age of participants remains optimal to examine those most likely to experience age‐related cognitive decline, a well‐established precursor of neurodegenerative disease (see supporting information). However, those included with useable retinal images were younger and healthier, which might not reflect the full spectrum of cognitive status. It is noteworthy that the cognitive tests used to measure cognitive decline were specifically developed for UK Biobank with unknown validity and test–retest reliability.^34^, ^35^ However, the test performance of these cognitive tests,^35^ and their association with subsequent neurodegenerative diseases (particularly AD), have since been shown (see supporting information).

The ease, speed, and precision of AI‐derived vascular metrics generated from retinal imaging, and the strong definitive associations these show with cognitive status, may offer a biomarker to more accurately discriminate between those who subsequently develop neurodegenerative outcomes from those who do not. This biomarker could be enhanced by ongoing technological improvements in image capture, coupled with the additive value of OCT RNFL measures, in addition to deeper vascular OCT‐A assessment, which in time could become more routinely available.^18^ OCT RNFL thicknesses are available in UK Biobank but we are yet able to examine their associations with these RV measures. Examining the time course of structural changes in the retina associated with cognitive decline and how these relate to RV changes will provide further insight into the mechanism of disease. However, for now, relative to the high cost of hospital‐ or clinic‐based brain scanning or blood testing, CFP are low cost to acquire, non‐invasive, rapid, and scalable given availability within existing opticians and eye clinic health‐care pathways, maximizing population reach, providing the potential to screen for cognitive decline and intervene early to avert/delay age‐related neurodegenerative outcomes.^48^, ^49^

## AUTHOR CONTRIBUTIONS

All authors contributed to this manuscript. Christopher G. Owen, Royce Shakespeare, Alicja R. Rudnicka, and Paul J. Foster designed the present study. Christopher G. Owen, Alicja R. Rudnicka, Sarah A. Barman, and Paul J. Foster raised funding. Royce Shakespeare, Christopher G. Owen, Alicja R. Rudnicka, Roshan Welikala, and Sarah A. Barman collected data for the study and undertook data management. Royce Shakespeare, Alicja R. Rudnicka, Roshan Welikala, and Sarah A. Barman analyzed the data. Christopher G. Owen and Royce Shakespeare wrote the first draft of the report, which all authors contributed to and critically appraised. Royce Shakespeare and Christopher G. Owen are responsible for data integrity and will act as guarantors.

## CONFLICT OF INTEREST STATEMENT

R.S.: None. A.R.R.: None. R.W.: None. S.A.B.: None. P.J.F.: None. C.G.O.: None. A.P.K.: Consultant or lecturer: Abbvie, Aerie, Allergan, Google Health, Heidelberg Novartis, Reichert, Santen, Thea, Topcon. Author disclosures are available in the supporting information.

## CONSENT STATEMENT

All participants gave written, informed consent.

## ETHICS STATEMENT

The UK Biobank study was carried out following the principles of the Declaration of Helsinki and the Research Governance Framework for Health and Social Care. The UK Biobank study was approved by the North West Multi‐Centre Research Ethics Committee (11/NW/03820).

## Supporting information



Supporting Information

Supporting Information

## Data Availability

The data reported in this article are available via application to the UK Biobank to other researchers for purposes of reproducing the results or replicating the procedure.

## COLLABORATORS

Prof Naomi Allen—University of Oxford

Prof Tariq Aslam—The University of Manchester

Dr Denize Atan—University of Bristol

Prof Konstantinos Balaskas—Moorfields Eye Hospital

Prof Sarah Barman—Kingston University

Prof Jenny Barrett—University of Leeds

Prof Paul Bishop—The University of Manchester

Prof Graeme Black—The University of Manchester

Dr Tasanee Braithwaite—St Thomas' Hospital

Dr Roxana Carare—University of Southampton

Prof Usha Chakravarthy—Queen'S University Belfast

Dr Michelle Chan—Moorfields Eye Hospital

Dr Sharon Chua—Ucl Institute of Ophthalmology

Dr Annegret Dahlmann‐Noor—Moorfields Eye Hospital

Dr Alexander Day—Moorfields Eye Hospital

Dr Parul Desai—Moorfields Eye Hospital

Prof Bal Dhillon—University of Edinburgh

Prof Andrew Dick—University of Bristol

Dr Alexander Doney—University of Dundee

Dr Cathy Egan—Moorfields Eye Hospital

Prof Sarah Ennis—University of Southampton

Prof Paul Foster—Ucl Institute of Ophthalmology

Dr Marcus Fruttiger—Ucl Institute of Ophthalmology

Dr John Gallacher—University of Oxford

Prof David (Ted) Garway‐Heath—Ucl Institute of Ophthalmology

Dr Jane Gibson—University of Southampton

Prof Jeremy Guggenheim—Cardiff University

Prof Chris Hammond—King'S College London

Prof Alison Hardcastle—Ucl Institute of Ophthalmology

Prof Simon Harding—University of Liverpool

Dr Ruth Hogg—Queen'S University Belfast

Dr Pirro Hysi—King'S College London

Prof Pearse Keane—Ucl Institute of Ophthalmology

Prof Sir Peng Tee Khaw—Ucl Institute of Ophthalmology

Prof Anthony Khawaja—Moorfields Eye Hospital

Mr Gerassimos Lascaratos—Moorfields Eye Hospital

Dr Adam Lewandowski—University of Oxford

Dr Thomas Littlejohns—University of Oxford

Prof Andrew Lotery—University of Southampton

Dr Robert Luben—Ucl Institute of Ophthalmology

Prof Phil Luthert—Ucl Institute of Ophthalmology

Dr Tom Macgillivray—University of Edinburgh

Dr Sarah Mackie—University of Leeds

Dr Savita Madhusudhan—Royal Liverpool University Hospital

Dr Bernadette Mcguinness—Queen'S University Belfast

Dr Gareth Mckay—Queen'S University Belfast

Dr Martin Mckibbin—Leeds Teaching Hospitals Nhs Trust

Prof Tony Moore—Ucl Institute of Ophthalmology

Prof James Morgan—Cardiff University

Dr Eoin O'Sullivan—King'S College Hospital

Prof Richard Oram—University of Exeter

Prof Chris Owen—St George'S, University of London

Dr Praveen Patel—Moorfields Eye Hospital

Dr Euan Paterson—Queen'S University Belfast

Prof Tunde Peto—Queen'S University Belfast

Dr Axel Petzold—Ucl Institute of Neurology

Dr Nikolas Pontikos—Ucl Institute of Ophthalmology

Prof Jugnoo Rahi—Ucl Institute of Child Health

Prof Alicja Rudnicka—St George'S, University of London

Prof Naveed Sattar—University of Glasgow

Dr Jay Self—University of Southampton

Dr Panagiotis Sergouniotis—The University of Manchester

Prof Sobha Sivaprasad—Moorfields Eye Hospital

Prof David Steel—Newcastle University

Mrs Irene Stratton—Gloucestershire Hospitals Nhs Foundation Trust

Dr Nicholas Strouthidis—Moorfields Eye Hospital

Prof Cathie Sudlow—University of Edinburgh

Dr Zihan Sun—Ucl Institute of Ophthalmology

Dr Robyn Tapp—St George'S, University of London

Dr Dhanes Thomas—Moorfields Eye Hospital

Dr Mervyn Thomas—University of Leicester

Prof Emanuele Trucco—University of Dundee

Prof Adnan Tufail—Moorfields Eye Hospital

Dr Ananth Viswanathan—Moorfields Eye Hospital

Dr Veronique Vitart—University of Edinburgh

Dr Mike Weedon—University of Exeter

Dr Katie Williams—King'S College London

Prof Cathy Williams—University of Bristol

Prof Jayne Woodside—Queen'S University Belfast

Dr Max Yates—University of East Anglia

Dr Yalin Zheng—University of Liverpool
