# Supplementary material for: Retinal vasculometry associations with cognition status in UK Biobank
Source: Alzheimers Dement (Amst). 2025 Feb 24;17(1):e270087. doi: 10.1002/dad2.70087 (PMC11848343; doi:10.1002/dad2.70087)
Supplement: Supplementary file 1 — Supporting Information [file DAD2-17-e270087-s002.docx]

**Supplemental material**

**eTable 1: Description of UK Biobank cognition tests used in PCA to derive G4, the first unrotated component generated.**

| **Test** | **Alpha numeric code for test in UK Biobank** | **Description** |
| --- | --- | --- |
| Pairs matching test | n_399_* | A visual memory test based on identifying matching pair of cards. Test comprised of three trials with the number of pairs to be matched increasing with each trial.  The number of errors made in a trial were summed up as the score.  Only the second trial (12 cards, 6 pairs) was used in PCA. The first round was easy and only a subsample of participants completed the third trial |
| Reaction Time test | n_20023_* | Test designed to evaluate the reaction time of participants by measuring the time taken to identify cards with matching symbols. The score is the mean time in milliseconds. |
| Prospective Memory Test | n_20018_* | A single trial test where participants were provided instructions to complete a task before conducting all the other cognitive tests and the trial itself was introduced after completing the other cognitive tests. The score for this task is dichotomous. (1 if participant successfully completed the task.) |
| Fluid Intelligence Test | n_20016_* | Number of multiple-choice questions answered correctly in two mins. (Maximum 13) |

Where ‘_*’ denotes baseline or follow-up, and which phase of measure.

**eTable 2: Eigenvalues for computed principal components.**

| **Component** | **Eigenvalue** | **Difference** | **Proportion** | **Cumulative** |
| --- | --- | --- | --- | --- |
| **1** | 1.53128 | .634039 | 0.3828 | 0.3828 |
| **2** | .897238 | .0271713 | 0.2243 | 0.6071 |
| **3** | .870067 | .16865 | 0.2175 | 0.8246 |
| **4** | .701417 | - | 0.1754 | 1.0000 |

**eTable 3: Factor loading for principal component analysis of cognitive tests.**

| Test | Unrotated PC1 | Unrotated PC2 | Unrotated PC3 | Unrotated PC4 |
| --- | --- | --- | --- | --- |
| Pairs matching | -0.4371 | 0.6042 | -0.6374 | 0.1939 |
| Reaction time | -0.4357 | 0.4632 | 0.7661 | 0.0925 |
| Fluid Intelligence | 0.5848 | 0.2638 | 0.0810 | 0.7628 |
| Prospective memory | 0.5263 | 0.5922 | 0.0149 | -0.6100 |

**eTable 4: Difference in retinal vasculometry characteristics per SD increase in general cognition score (G4) and other covariates from multivariable regression models with different levels of adjustment.**

|  | **Model 1** | | **Model 2** | | **Model 3** | | **Model 4** | |
| --- | --- | --- | --- | --- | --- | --- | --- | --- |
|  | **Difference (95% CI)** | **p-value** | **Difference (95% CI)** | **p-value** | **Difference (95% CI)** | **p-value** | **Difference (95% CI)** | **p-value** |
| **Arteriolar width** |  |  |  |  |  |  |  |  |
| *per SD in G4* | 0.16 (0.09,0.22) | 1.49E^-06^ | 0.09 (0.03,0.15) | 0.01 | 0.09 (0.02,0.16) | 0.01 | 0.10 (0.02,0.18) | 0.01 |
| *per decade in age* | -0.81 (-0.89,-0.74) | 4.44E^-96^ | -0.61 (-0.69,-0.53) | 3.58E^-52^ | -0.06 (-0.15,0.03) | 0.19 | -0.02 (-0.13,0.08) | 0.66 |
| *Sex (female)* | -0.30 (-0.42,-0.19) | 4.91E^-07^ | 1.39 (1.22,1.57) | 5.95E^-57^ | 0.87 (0.67,1.06) | 1.25E^-18^ | 0.88 (0.65,1.10) | 2.02E^-14^ |
| *per 10 cm in height* |  |  | 1.27 (1.18,1.37) | 1.06E^-153^ | 1.18 (1.07,1.28) | 5.69E^-111^ | 1.23 (1.11,1.35) | 4.34E^-90^ |
| *BMI* |  |  |  |  | -0.02 (-0.04,-0.01) | 0.01 | -0.02 (-0.03,0.00) | 0.1 |
| *HbA1c mmol/mol* |  |  |  |  | 0.04 (0.02,0.05) | 3.24E-10 | 0.03 (0.02,0.05) | 1.42E^-05^ |
| *per 10 mmHg in SBP* |  |  |  |  | -0.85 (-0.89,-0.81) | < 10^-323^ | -0.92 (-0.97,-0.87) | < 10^-323^ |
| *cholesterol mmol/L* |  |  |  |  | -0.09 (-0.15,-0.03) | 4.36E^-03^ | -0.05 (-0.13,0.02) | 0.16 |
| *triglycerides mmol/L* |  |  |  |  | 0.08 (0.01,0.15) | 0.04 | 0.07 (-0.02,0.16) | 0.11 |
| **Venular width** |  |  |  |  |  |  |  |  |
| *per SD in G4* | 0.36  (0.26,0.47) | 1.48E^-11^ | 0.38 (0.28,0.49) | 2.34E^-12^ | 0.38  (0.26,0.49) | 4.31E^-10^ | 0.31  (0.17,0.45) | 1.71E-^05^ |
| *per decade in age* | 0.68  (0.55,0.81) | 1.70E^-25^ | 0.82 (0.69,0.95) | 1.54E^-34^ | 0.9  (0.74,1.05) | 4.67E^-30^ | 0.9  (0.71,1.08) | 4.09E^-22^ |
| *Sex (female)* | -0.77  (-0.97,-0.57) | 2.56E^-14^ | 0.07  (-0.22,0.36) | 0.63 | 0.16  (-0.17,0.49) | 0.35 | 0.33  (-0.06,0.72) | 0.09 |
| *per 10 cm in height* |  |  | 0.57  (0.41,0.73) | 1.10E^-12^ | 0.6  (0.43,0.78) | 1.50E^-11^ | 0.64  (0.43,0.85) | 1.30E^-09^ |
| *BMI* |  |  |  |  | 0.1  (0.08,0.13) | 1.29E^-15^ | 0.11  (0.08,0.14) | 1.88E^-11^ |
| *HbA1c mmol/mol* |  |  |  |  | 0.03  (0.01,0.05) | 8.10E^-04^ | 0.04  (0.01,0.07) | 2.46E^-03^ |
| *per 10 mmHg in SBP* |  |  |  |  | -0.29  (-0.36,-0.23) | 1.95E^-18^ | -0.29  (-0.37,-0.21) | 3.86E^-12^ |
| *cholesterol mmol/L* |  |  |  |  | 0.02  (-0.08,0.12) | 0.71 | -0.04  (-0.17,0.08) | 0.51 |
| *triglycerides mmol/L* |  |  |  |  | 0.35  (0.22,0.47) | 1.01E^-07^ | 0.4  (0.25,0.56) | 3.80E^-07^ |
| *Interaction (G4 and age)* | -0.4  (-0.52,-0.28) | 5.38E^-11^ | -0.41 (-0.53,-0.29) | 1.51E^-11^ | -0.44  (-0.57,-0.31) | 9.44E^-11^ | -0.4  (-0.55,-0.24) | 7.54E^-07^ |
| **Arteriolar tortuosity**† |  |  |  |  |  |  |  |  |
| *per SD in G4* | 0.48 (0.06,0.89) | 0.03 | 0.56 (0.14,0.98) | 0.01 | 0.67 (0.20,1.14) | 0.01 | 0.83 (0.27,1.39) | 3.48E^-03^ |
| *per decade in age* | 2.25 (1.74,2.77) | 2.59E^-18^ | 2.31 (1.79,2.84) | 4.04E^-18^ | 1.18 (0.56,1.80) | 1.90E^-04^ | 0.89 (0.16,1.61) | 0.02 |
| *Sex (female)* | 3.93 (3.13,4.74) | 1.77E^-22^ | 3.54 (2.38,4.72) | 1.48E^-09^ | 4.66 (3.30,6.03) | 7.43E^-12^ | 4.82 (3.22,6.44) | 1.77E^-09^ |
| *per 10 cm in height* |  |  | -0.33 (-0.94,0.29) | 0.3 | -0.22 (-0.91,0.48) | 0.54 | -0.32 (-1.14,0.50) | 0.44 |
| *BMI* |  |  |  |  | -0.15 (-0.25,-0.05) | 2.84E^-03^ | -0.31 (-0.44,-0.18) | 1.70E^-06^ |
| *HbA1c mmol/mol* |  |  |  |  | 0.13 (0.05,0.20) | 8.19E^-04^ | 0.09 (-0.01,0.20) | 0.08 |
| *per 10 mmHg in SBP* |  |  |  |  | 1.32 (1.05,1.58) | 6.47E^-23^ | 1.18 (0.85,1.51) | 1.40E^-12^ |
| *cholesterol mmol/L* |  |  |  |  | -0.04 (-0.45,0.37) | 0.84 | 0.24 (-0.27,0.74) | 0.36 |
| *triglycerides mmol/L* |  |  |  |  | 0.62 (0.12,1.13) | 0.02 | 0.72 (0.11,1.34) | 0.02 |
| ***Venular tortuosity***† |  |  |  |  |  |  |  |  |
| *per SD in G4* | -0.68 (-0.93,-0.44) | 6.92E^-08^ | -0.59 (-0.84,-0.34) | 4.65E^-06^ | -0.39 (-0.67,-0.11) | 0.01 | -0.31 (-0.63,0.02) | 0.06 |
| *per decade in age* | 2.27 (1.96,2.58) | 1.21E^-48^ | 2.37 (2.05,2.68) | 1.86E^-49^ | 1.92 (1.55,2.30) | 1.90E^-24^ | 1.78 (1.35,2.21) | 4.14E^-16^ |
| *Sex (female)* | 1.25 (0.78,1.72) | 1.67E^-07^ | 1.16 (0.48,1.85) | 8.17E^-04^ | 1.97 (1.18,2.77) | 8.09E^-07^ | 1.74 (0.83,2.67) | 1.82E^-04^ |
| *per 10 cm in height* |  |  | -0.11 (-0.48,0.27) | 0.58 | 0.14 (-0.27,0.56) | 0.50 | 0.17 (-0.31,0.66) | 0.48 |
| *BMI* |  |  |  |  | 0.42 (0.36,0.48) | 4.90E^-42^ | 0.29 (0.21,0.36) | 1.27E^-13^ |
| *HbA1c mmol/mol* |  |  |  |  | 0.13 (0.09,0.18) | 5.48E^-09^ | 0.10 (0.04,0.16) | 1.84E^-03^ |
| *per 10 mmHg in SBP* |  |  |  |  | 0.48 (0.33,0.64) | 1.30E^-09^ | 0.42 (0.22,0.61) | 2.33E^-05^ |
| *cholesterol mmol/L* |  |  |  |  | -0.43 (-0.67,-0.19) | 5.32E^-04^ | -0.11 (-0.40,0.19) | 0.48 |
| *triglycerides mmol/L* |  |  |  |  | -0.46 (-0.76,-0.16) | 2.86E^-03^ | -0.54 (-0.90,-0.18) | 3.26E^-03^ |
| **Arteriolar area** |  |  |  |  |  |  |  |  |
| *per SD in G4* | 0.01 (0.00,0.02) | 2.01E^-03^ | 0.01 (0.00,0.01) | 0.04 | 0.01 (0.00,0.01) | 0.16 | 0.53 (-0.32,1.38)* | 0.22 |
| *per decade in age* | -0.36 (-0.37,-0.35) | < 10^-323^ | -0.35 (-0.36,-0.35) | < 10^-323^ | -0.31 (-0.32,-0.31) | < 10^-323^ | -0.32 (-0.33,-0.31) | < 10^-323^ |
| *Sex (female)* | -0.05 (-1.23,1.13) * | 0.93 | 0.08 (0.06,0.09) | 8.27E^-18^ | 0.02 (0.00,0.04) | 0.02 | 0.02 (-0.01,0.04) | 0.18 |
| *per 10 cm in height* |  |  | 0.06 (0.05,0.07) | 4.60E^-32^ | 0.05 (0.04,0.06) | 2.39E^-18^ | 0.05 (0.03,0.06) | 1.47E^-13^ |
| *BMI* |  |  |  |  | -0.01 (-0.01,-0.01) | 1.73E^-33^ | -0.01 (-0.01,-0.01) | 2.73E^-19^ |
| *HbA1c mmol/mol* |  |  |  |  | 0.01 (-0.10,0.12)* | 0.91 | 0.08 (-0.08,0.24)* | 0.32 |
| *per 10 mmHg in SBP* |  |  |  |  | -0.06 (-0.06,-0.05) | 2.96E^-173^ | -0.06 (-0.06,-0.05) | 7.01E^-117^ |
| *cholesterol mmol/L* |  |  |  |  | 0.28 (-0.33,0.89)* | 0.37 | -0.09 (-0.86,0.68)* | 0.82 |
| *triglycerides mmol/L* |  |  |  |  | -0.36 (-1.12,0.40)* | 0.35 | 0.25 (-0.69,1.18)* | 0.6 |
| **Venular area** |  |  |  |  |  |  |  |  |
| *per SD in G4* | 0.04  (0.03,0.05) | 9.91E^-27^ | 0.03 (0.03,0.04) | 3.94E^-19^ | 0.03  (0.03,0.04) | 1.15E^-16^ | 0.04  (0.03,0.05) | 3.70E^-16^ |
| *per decade in age* | -0.07  (-0.08,-0.06) | 6.53E^-54^ | -0.05  (-0.06,-0.04) | 2.38E^-33^ | -0.04  (-0.05,-0.03) | 2.94E^-14^ | -0.04  (-0.05,-0.03) | 8.81E^-12^ |
| *Sex (female)* | -0.21  (-0.22,-0.2) | 1.47E^-213^ | -0.08  (-0.1,-0.06) | 2.35E^-17^ | -0.08  (-0.1,-0.06) | 9.04E^-13^ | -0.07  (-0.1,-0.05) | 3.57E^-08^ |
| *per 10 cm in height* |  |  | 0.1  (0.08,0.11) | 1.80E^-70^ | 0.1  (0.09,0.11) | 1.67E^-59^ | 0.1  (0.08,0.11) | 3.27E^-42^ |
| *BMI* |  |  |  |  | 0.01  (0.01,0.01) | 1.48E^-24^ | 0.01  (0.01,0.01) | 2.23E^-12^ |
| *HbA1c mmol/mol* |  |  |  |  | 0.09  (-0.03,0.22)* | 0.15 | 0.09  (-0.09,0.26)* | 0.33 |
| *per 10 mmHg in SBP* |  |  |  |  | -0.02  (-0.03,-0.02) | 2.17E^-27^ | -0.02  (-0.03,-0.02) | 1.14E^-16^ |
| *cholesterol mmol/L* |  |  |  |  | 0.01  (0,0.02) | 2.93E^-03^ | 0.01  (0,0.02) | 0.01 |
| *triglycerides mmol/L* |  |  |  |  | 0.01  (0,0.02) | 0.03 | 0.01  (0,0.02) | 0.02 |
| *Interaction (G4 and age)* | 0.02  (0.01,0.03) | 2.36E^-08^ | 0.02 (0.01,0.03) | 2.47E^-08^ | 0.02  (0.01,0.03) | 1.20E^-05^ | 0.02  (0.01,0.03) | 8.87E^-05^ |
| **1/ (Arteriolar SD(µm))*** |  |  |  |  |  |  |  |  |
| *per SD in G4* | -0.06(-0.08,-0.04) | 5.48E^-08^ | -0.06 (-0.08,-0.04) | 5.66E^-08^ | -0.06 (-0.08,-0.04) | 1.82E^-07^ | -0.06 (-0.09,-0.03) | 1.94E^-05^ |
| *per decade in age* | -0.93 (-0.95,-0.90) | < 10^-323^ | -0.93 (-0.96,-0.91) | < 10^-323^ | -0.85 (-0.88,-0.82) | < 10^-323^ | -0.87 (-0.91,-0.84) | < 10^-323^ |
| *Sex (female)* | 0.08 (0.04,0.12) | 3.06E^-05^ | 0.03 (-0.02,0.09) | 0.23 | -0.05 (-0.12,0.01) | 0.10 | -0.08 (-0.16,0.00) | 0.04 |
| *per 10 cm in height* |  |  | -0.03 (-0.06,0.00) | 0.04 | -0.05 (-0.08,-0.02) | 3.34E^-03^ | -0.04 (-0.08,0.00) | 0.04 |
| *BMI* |  |  |  |  | -0.01 (-0.01,0.00) | 1.48E^-03^ | -0.01 (-0.02,-0.01) | 2.21E^-04^ |
| *HbA1c mmol/mol* |  |  |  |  | 0.00 (-0.01,0.00) | 0.21 | 0.00 (-0.01,0.00) | 0.17 |
| *per 10 mmHg in SBP* |  |  |  |  | -0.11 (-0.12,-0.09) | 9.37E^-62^ | -0.11 (-0.13,-0.09) | 2.16E^-41^ |
| *cholesterol mmol/L* |  |  |  |  | -0.02 (-0.04,0.00) | 0.10 | 0.00 (-0.03,0.02) | 0.89 |
| *triglycerides mmol/L* |  |  |  |  | 0.00 (-0.03,0.02) | 0.90 | -0.01 (-0.04,0.02) | 0.6 |
| **1/ (Venular SD(µm))*** |  |  |  |  |  |  |  |  |
| *per SD in G4* | -0.05 (-0.08,-0.03) | 6.04E^-06^ | -0.05 (-0.08,-0.03) | 5.53E-06 | -0.05 (-0.08,-0.03) | 7.15E-05 | -0.05 (-0.08,-0.02) | 1.00E^-03^ |
| *per decade in age* | -1.03 (-1.06,-1.00) | < 10^-323^ | -1.04 (-1.07,-1.02) | < 10^-323^ | -1.04 (-1.07,-1.00) | < 10^-323^ | -1.04 (-1.08,-1.00) | < 10^-323^ |
| *Sex (female)* | 0.00 (-0.04,0.05) | 0.93 | -0.10 (-0.16,-0.04) | 2.01E-03 | -0.13 (-0.21,-0.06) | 3.15E^-04^ | -0.18 (-0.27,-0.10) | 3.85E^-05^ |
| *per 10 cm in height* |  |  | -0.07  (-0.11,-0.04) | 4.16E-05 | -0.09 (-0.12,-0.05) | 1.72E^-05^ | -0.10 (-0.15,-0.05) | 2.22E^-05^ |
| *BMI* |  |  |  |  | -0.02 (-0.02,-0.01) | 4.36E^-08^ | -0.02 (-0.02,-0.01) | 2.23E^-06^ |
| *HbA1c mmol/mol* |  |  |  |  | -0.01 (-0.01,0.00) | 1.49E^-04^ | -0.01 (-0.01,0.00) | 0.02 |
| *per 10 mmHg in SBP* |  |  |  |  | 0.02 (0.00,0.03) | 0.02 | 0.02 (0.00,0.04) | 0.02 |
| *cholesterol mmol/L* |  |  |  |  | 0.01 (-0.02,0.03) | 0.51 | 0.00 (-0.03,0.03) | 0.90 |
| *triglycerides mmol/L* |  |  |  |  | 0.00 (-0.03,0.03) | 0.87 | 0.00 (-0.04,0.03) | 0.96 |

† - Coefficients as % change for log transformed target variables. * - Coefficients multiplied by 100. Model 1 - adjusted for age, sex, ethnicity and UK Biobank centre. Model 2 - Model 1 adjustment, with adjustment for smoking, Townsend deprivation index and height. Model 3 – Model 2 adjustment, with adjustment for BMI, HbA1c, systolic BP, total cholesterol and triacylglycerols. Model 4 – Same as Model 3 but excluding persons with self-reported history of heart attack, stroke, hypertension or on medication for hypertension.

**eTable 5: Difference in retinal vasculometry characteristics per SD increase in general cognition score (G4) and other covariates from multivariable regression models with different levels of adjustment (including educational qualification).**

|  | **Model 1** | | **Model 2** | | **Model 3** | | **Model 4** | |
| --- | --- | --- | --- | --- | --- | --- | --- | --- |
|  | **Difference (95% CI)** | **p-value** | **Difference (95% CI)** | **p-value** | **Difference (95% CI)** | **p-value** | **Difference (95% CI)** | **p-value** |
| **Arteriolar width** |  |  |  |  |  |  |  |  |
| *per SD in G4* | 0.13 (0.06,0.19) | 1.77E^-04^ | 0.07 (0.00,0.14) | 0.04 | 0.09 (0.02,0.16) | 0.02 | 0.10 (0.01,0.18) | 0.02 |
| *per decade in age* | -0.81 (-0.89,-0.73) | 1.37E^-92^ | -0.61 (-0.69,-0.53) | 1.36E^-50^ | -0.06 (-0.15,0.03) | 0.18 | -0.02 (-0.13,0.08) | 0.67 |
| *Sex (female)* | -0.31 (-0.43,-0.19) | 2.68E^-07^ | 1.38 (1.20,1.55) | 3.01E^-55^ | 0.85 (0.66,1.05) | 4.04E^-18^ | 0.87 (0.64,1.09) | 4.88E^-14^ |
| *per 10 cm in height* |  |  | 1.26 (1.17,1.36) | 9.11E^-151^ | 1.17 (1.07,1.28) | 4.34E^-110^ | 1.23 (1.11,1.35) | 2.20E^-89^ |
| *BMI* |  |  |  |  | -0.02 (-0.04,-0.01) | 6.46E^-03^ | -0.02 (-0.03,0.00) | 0.12 |
| *HbA1c mmol/mol* |  |  |  |  | 0.04 (0.02,0.05) | 3.01E^-10^ | 0.03 (0.02,0.05) | 1.40E^-05^ |
| *per 10 mmHg in SBP* |  |  |  |  | -0.85 (-0.89,-0.81) | <10^-323^ | -0.92 (-0.97,-0.87) | <10^-323^ |
| *cholesterol mmol/L* |  |  |  |  | -0.09 (-0.15,-0.03) | 4.30E^-03^ | -0.05 (-0.13,0.02) | 0.16 |
| *triglycerides mmol/L* |  |  |  |  | 0.08 (0.00,0.15) | 0.04 | 0.07 (-0.02,0.16) | 0.12 |
| **Venular width** |  |  |  |  |  |  |  |  |
| *per SD in G4* | 0.37 (0.26,0.48) | 9.06E^-11^ | 0.37  (0.26,0.48) | 1.01E^-10^ | 0.35  (0.22,0.47) | 3.32E^-08^ | 0.28  (0.14,0.43) | 1.72E^-04^ |
| *per decade in age* | 0.69 (0.56,0.81) | 2.54E^-25^ | 0.84  (0.70,0.97) | 8.91E^-35^ | 0.92  (0.76,1.07) | 2.10E^-30^ | 0.92  (0.74,1.11) | 7.53E^-23^ |
| *Sex (female)* | -0.78 (-0.98,-0.59) | 8.33E^-15^ | 0.05 (-0.24,0.33) | 0.75 | 0.13  (-0.20,0.45) | 0.45 | 0.30  (-0.08,0.69) | 0.12 |
| *per 10 cm in height* |  |  | 0.57 (0.41,0.73) | 1.99E^-12^ | 0.59  (0.42,0.77) | 3.73E^-11^ | 0.63  (0.43,0.84) | 2.24E^-09^ |
| *BMI* |  |  |  |  | 0.11  (0.08,0.13) | 5.75E^-16^ | 0.11  (0.08,0.14) | 1.30E^-11^ |
| *HbA1c mmol/mol* |  |  |  |  | 0.03  (0.01,0.05) | 7.62E^-04^ | 0.04  (0.01,0.07) | 2.42E^-03^ |
| *per 10 mmHg in SBP* |  |  |  |  | -0.29  (-0.36,-0.23) | 2.42E^-18^ | -0.29  (-0.37,-0.21) | 4.89E^-12^ |
| *cholesterol mmol/L* |  |  |  |  | 0.02  (-0.08,0.12) | 0.73 | -0.04  (-0.17,0.08) | 0.50 |
| *triglycerides mmol/L* |  |  |  |  | 0.35  (0.22,0.47) | 9.66E^-08^ | 0.40  (0.25,0.56) | 3.91E^-07^ |
| *Interaction (G4 and age)* | -0.41  (-0.53,-0.29) | 2.98E^-11^ | -0.42  (-0.54,-0.30) | 4.64E^-12^ | -0.45  (-0.59,-0.32) | 2.36E^-11^ | -0.42  (-0.57,-0.26) | 2.43E^-07^ |
| **Arteriolar tortuosity**† |  |  |  |  |  |  |  |  |
| *per SD in G4* | 0.35 (-0.09,0.78) | 0.12 | 0.41 (-0.03,0.86) | 0.06 | 0.54 (0.05,1.03) | 0.03 | 0.67 (0.10,1.26) | 2.25E^-02^ |
| *per decade in age* | 2.36 (1.85,2.89) | 1.76E^-19^ | 2.44 (1.90,2.98) | 2.03E^-19^ | 1.30 (0.67,1.93) | 4.51E^-05^ | 0.96 (0.23,1.70) | 9.69E^-03^ |
| *Sex (female)* | 3.92 (3.11,4.73) | 3.37E^-22^ | 3.47 (2.31,4.65) | 3.47E^-09^ | 4.58 (3.22,5.95) | 1.73E^-11^ | 4.73 (3.13,6.35) | 3.61E^-09^ |
| *per 10 cm in height* |  |  | -0.37 (-0.99,0.25) | 0.24 | -0.26 (-0.95,0.44) | 0.47 | -0.38 (-1.19,0.44) | 0.37 |
| *BMI* |  |  |  |  | -0.15 (-0.25,-0.05) | 3.69E^-03^ | -0.30 (-0.43,-0.18) | 2.99E^-06^ |
| *HbA1c mmol/mol* |  |  |  |  | 0.13 (0.05,0.20) | 7.79E^-04^ | 0.09 (-0.01,0.20) | 0.08 |
| *per 10 mmHg in SBP* |  |  |  |  | 1.32 (1.06,1.59) | 4.52E^-23^ | 1.19 (0.87,1.52) | 8.58E^-13^ |
| *cholesterol mmol/L* |  |  |  |  | -0.05 (-0.46,0.36) | 0.80 | 0.23 (-0.27,0.74) | 0.36 |
| *triglycerides mmol/L* |  |  |  |  | 0.63 (0.12,1.14) | 0.02 | 0.73 (0.11,1.35) | 0.02 |
| ***Venular tortuosity***† |  |  |  |  |  |  |  |  |
| *per SD in G4* | -0.46 (-0.72,-0.20) | 5.95E^-04^ | -0.40 (-0.66,-0.14) | 2.90E^-03^ | -0.26 (-0.55,0.03) | 0.08 | -0.24 (-0.58,0.10) | 0.17 |
| *per decade in age* | 2.20 (1.89,2.51) | 1.37E^-44^ | 2.31 (1.99,2.63) | 4.04E^-46^ | 1.88 (1.50,2.25) | 7.44E^-23^ | 1.75 (1.32,2.19) | 2.10E^-15^ |
| *Sex (female)* | 1.29 (0.81,1.76) | 8.19E^-08^ | 1.29 (0.60,1.98) | 2.28E^-04^ | 2.04 (1.25,2.83) | 3.70E^-07^ | 1.79 (0.87,2.72) | 1.22E^-04^ |
| *per 10 cm in height* |  |  | -0.03 (-0.40,0.35) | 0.89 | 0.19 (-0.23,0.61) | 0.37 | 0.20 (-0.28,0.69) | 0.42 |
| *BMI* |  |  |  |  | 0.41 (0.35,0.47) | 1.45E^-40^ | 0.28 (0.21,0.36) | 3.09E^-13^ |
| *HbA1c mmol/mol* |  |  |  |  | 0.13 (0.09,0.18) | 5.91E^-09^ | 0.10 (0.04,0.16) | 1.86E^-03^ |
| *per 10 mmHg in SBP* |  |  |  |  | 0.48 (0.32,0.63) | 2.33E^-09^ | 0.41 (0.22,0.60) | 2.99E^-05^ |
| *cholesterol mmol/L* |  |  |  |  | -0.42 (-0.67,-0.18) | 6.45E^-04^ | -0.11 (-0.40,0.19) | 0.49 |
| *triglycerides mmol/L* |  |  |  |  | -0.46 (-0.76,-0.16) | 2.53E^-03^ | -0.54 (-0.90,-0.18) | 3.19E^-03^ |
| **Arteriolar area** |  |  |  |  |  |  |  |  |
| *per SD in G4* | 0.01  (0.00,0.01) | 3.36E^-02^ | 0.00  (0.00,0.01) | 0.19 | 0.01 (0.00,0.01) | 0.14 | 0.72 (-0.16,1.60)* | 0.11 |
| *per decade in age* | -0.36(-0.37,-0.35) | <10^-323^ | -0.35(-0.36,-0.35) | <10^-323^ | -0.31 (-0.32,-0.31) | <10^-323^ | -0.32 (-0.33,-0.31) | <10^-323^ |
| *Sex (female)* | -0.03(-1.21,1.16) * | 0.96 | 0.07(0.06,0.09) | 2.21E^-17^ | 0.02 (0.00,0.04) | 0.02 | 0.02 (-0.01,0.04) | 0.16 |
| *per 10 cm in height* |  |  | 0.06(0.05,0.07) | 7.05E^-31^ | 0.05 (0.04,0.06) | 2.26E^-18^ | 0.05 (0.04,0.06) | 7.48E^-14^ |
| *BMI* |  |  |  |  | -0.01 (-0.01,-0.01) | 1.95E^-33^ | -0.01 (-0.01,-0.01) | 1.40E^-19^ |
| *HbA1c mmol/mol* |  |  |  |  | 0.01 (-0.11,0.12)* | 0.91 | 0.08 (-0.08,0.24)* | 0.32 |
| *per 10 mmHg in SBP* |  |  |  |  | -0.06 (-0.06,-0.05) | 3.72E^-173^ | -0.06 (-0.06,-0.05) | 2.71E^-117^ |
| *cholesterol mmol/L* |  |  |  |  | 0.28 (-0.33,0.90)* | 0.36 | -0.09 (-0.85,0.68)* | 0.83 |
| *triglycerides mmol/L* |  |  |  |  | -0.36 (-1.12,0.40)* | 0.35 | 0.24 (-0.69,1.18)* | 0.61 |
| **Venular area** |  |  |  |  |  |  |  |  |
| *per SD in G4* | 0.03  (0.02,0.04) | 7.91E^-17^ | 0.03  (0.02,0.03) | 2.55E^-12^ | 0.03  (0.02,0.04) | 1.28E^-10^ | 0.03 (0.02,0.04) | 2.27E^-10^ |
| *per decade in age* | -0.07  (-0.07,-0.06) | 5.46E^-50^ | -0.05  (-0.06,-0.04) | 7.40E^-31^ | -0.04  (-0.05,-0.03) | 4.29E^-13^ | -0.04 (-0.05,-0.03) | 7.39E^-11^ |
| *Sex (female)* | -0.21  (-0.22,-0.20) | 1.70E^-213^ | -0.09  (-0.11,-0.07) | 1.39E^-18^ | -0.08  (-0.10,-0.06) | 1.50E^-13^ | -0.08 (-0.10,-0.05) | 6.79E^-09^ |
| *per 10 cm in height* |  |  | 0.09  (0.08,0.10) | 1.35E^-66^ | 0.09  (0.08,0.11) | 1.68E^-56^ | 0.09 (0.08,0.11) | 6.80E^-40^ |
| *BMI* |  |  |  |  | 0.01  (0.01,0.01) | 2.31E^-26^ | 0.01 (0.01,0.01) | 1.64E^-13^ |
| *HbA1c mmol/mol* |  |  |  |  | 0.09  (-0.03,0.22)* | 0.15 | 0.09  (-0.09,0.26)* | 0.33 |
| *per 10 mmHg in SBP* |  |  |  |  | -0.02  (-0.03,-0.02) | 1.39E^-26^ | -0.02  (-0.03,-0.02) | 5.86E^-16^ |
| *cholesterol mmol/L* |  |  |  |  | 0.01  (0.00,0.02) | 3.64E^-03^ | 0.01  (0.00,0.02) | 0.01 |
| *triglycerides mmol/L* |  |  |  |  | 0.01  (0.00,0.02) | 0.02 | 0.01  (0.00,0.02) | 0.02 |
| *Interaction (G4 and age)* | 0.02  (0.01,0.03) | 2.15E^-07^ | 0.02  (0.01,0.03) | 2.10E^-07^ | 0.02  (0.01,0.03) | 7.10E^-05^ | 0.02  (0.01,0.03) | 4.93E^-04^ |
| **1/ (Arteriolar SD(µm))** * |  |  |  |  |  |  |  |  |
| *per SD in G4* | -0.05 (-0.07,-0.03) | 1.67E^-05^ | -0.05 (-0.07,-0.03) | 1.36E^-05^ | -0.05 (-0.07,-0.02) | 9.35E^-05^ | -0.05 (-0.08,-0.02) | 1.14E^-03^ |
| *per decade in age* | -0.93 (-0.96,-0.91) | <10^-323^ | -0.94 (-0.97,-0.91) | <10^-323^ | -0.86 (-0.89,-0.83) | <10^-323^ | -0.88 (-0.91,-0.84) | <10^-323^ |
| *Sex (female)* | 0.08 (0.04,0.12) | 1.96E^-05^ | 0.04 (-0.02,0.09) | 0.16 | -0.05 (-0.11,0.02) | 0.14 | -0.08 (-0.15,0.00) | 0.05 |
| *per 10 cm in height* |  |  | -0.03 (-0.06,0.00) | 0.07 | -0.05 (-0.08,-0.01) | 7.71E^-03^ | -0.04 (-0.08,0.00) | 0.07 |
| *BMI* |  |  |  |  | -0.01 (-0.01,0.00) | 6.51E^-04^ | -0.01 (-0.02,-0.01) | 1.21E^-04^ |
| *HbA1c mmol/mol* |  |  |  |  | 0.00 (-0.01,0.00) | 0.20 | 0.00 (-0.01,0.00) | 0.17 |
| *per 10 mmHg in SBP* |  |  |  |  | -0.11 (-0.12,-0.10) | 1.52E^-62^ | -0.11 (-0.13,-0.10) | 4.21E^-42^ |
| *cholesterol mmol/L* |  |  |  |  | -0.02 (-0.04,0.00) | 0.11 | 0.00 (-0.03,0.02) | 0.91 |
| *triglycerides mmol/L* |  |  |  |  | 0.00 (-0.03,0.02) | 0.86 | -0.01 (-0.04,0.02) | 0.58 |
| **1/ (Venular SD(µm))** * |  |  |  |  |  |  |  |  |
| *per SD in G4* | -0.04 (-0.07,-0.02) | 7.08E^-04^ | -0.04 (-0.07,-0.02) | 6.25E^-04^ | -0.04 (-0.07,-0.01) | 5.36E^-03^ | -0.04 (-0.07,-0.01) | 1.79E^-02^ |
| *per decade in age* | -1.03 (-1.06,-1.00) | <10^-323^ | -1.05 (-1.08,-1.02) | <10^-323^ | -1.04 (-1.08,-1.01) | <10^-323^ | -1.04 (-1.09,-1.00) | <10-^323^ |
| *Sex (female)* | 0.00 (-0.04,0.05) | 0.91 | -0.09 (-0.16,-0.03) | 3.89E^-03^ | -0.13 (-0.20,-0.06) | 5.42E^-04^ | -0.18 (-0.27,-0.09) | 6.57E^-05^ |
| *per 10 cm in height* |  |  | -0.07 (-0.10,-0.03) | 1.31E^-04^ | -0.08 (-0.12,-0.04) | 5.42E^-05^ | -0.10 (-0.14,-0.05) | 5.34E^-05^ |
| *BMI* |  |  |  |  | -0.02 (-0.02,-0.01) | 1.19E^-08^ | -0.02 (-0.03,-0.01) | 1.15E^-06^ |
| *HbA1c mmol/mol* |  |  |  |  | -0.01 (-0.01,0.00) | 1.42E^-04^ | -0.01 (-0.01,0.00) | 0.02 |
| *per 10 mmHg in SBP* |  |  |  |  | 0.02 (0.00,0.03) | 0.02 | 0.02 (0.00,0.04) | 0.03 |
| *cholesterol mmol/L* |  |  |  |  | 0.01 (-0.01,0.03) | 0.47 | 0.00 (-0.03,0.03) | 0.92 |
| *triglycerides mmol/L* |  |  |  |  | 0.00 (-0.03,0.03) | 0.83 | 0.00 (-0.04,0.03) | 0.94 |

† - Coefficients as % change for log transformed target variables. * - Coefficients multiplied by 100. Model 1 - adjusted for age, sex, ethnicity, educational qualification, and UK Biobank centre. Model 2 - Model 1 adjustment, with adjustment for smoking, Townsend deprivation index and height. Model 3 – Model 2 adjustment, with adjustment for BMI, HbA1c, systolic BP, total cholesterol and triacylglycerols. Model 4 – Same as Model 3 but excluding persons with self-reported history of heart attack, stroke, hypertension or on medication for hypertension.

**eTable 6: Difference in retinal vasculometry characteristics per SD increase in general cognition score (G4) by ethnic group**

| **Vessel characteristic** | **White** | | **Black** | | **Asian** | |
| --- | --- | --- | --- | --- | --- | --- |
|  | **Difference (95% CI)** | **P-value** | **Difference (95% CI)** | **P-value** | **Difference (95% CI)** | **P-value** |
| **Arteriolar width (µm)** | 0.18(0.11,0.24) | 1.52E^-07^ | 0.32(-0.04,0.68) | 0.08 | 0.25(-0.11,0.61) | 0.17 |
| **Venular width (µm)** | 0.42(0.31,0.53) | 1.62E^-14^ | 0.93(0.07,1.78) | 0.03 | 1.63(0.84,2.41) | 4.86E^-05^ |
| **Arteriolar vessel Area (mm²)** | 0.01(0,0.02) | 4.19E^-03^ | 0.05(0.01,0.08) | 0.01 | 0.09(0.06,0.13) | 9.69E^-08^ |
| **Venular vessel area (mm²)** | 0.03(0.02,0.03) | 2.75E^-12^ | -0.04(-0.1,0.01) | 0.13 | -0.04(-0.1,0.02) | 0.16 |
| **1/ (Arteriolar SD(µm))*** | -0.09(-0.12, -0.07) | 1.80E^-18^ | -0.06(-0.17,0.04) | 0.25 | -0.05(-0.17,0.06) | 0.38 |
| **1/ (Venular SD(µm))*** | -0.08(-0.1, -0.05) | 1.40E^-10^ | -0.1(-0.23,0.04) | 0.16 | -0.18(-0.33, -0.04) | 0.01 |
| **Arteriolar tortuosity** † | 0.36(-0.08,0.79) | 0.11 | 1.11(-1.14,3.4) | 0.34 | 0.58(-1.63,2.85) | 0.61 |
| **Venular tortuosity** † | -0.89(-1.14, -0.63) | 7.55E^-12^ | 0.1(-1.55,1.78) | 0.91 | -0.01(-0.02,0.01) | 0.44 |

† - Coefficients as % change for log transformed target variables. * - Coefficients multiplied by 100. Model 1 - adjusted for age, sex and UK Biobank centre.

**eTable 7: Difference in retinal vasculometry characteristics per SD increase in fluid intelligence (FI) score and other covariates from multivariable regression models with different levels of adjustment.**

|  | **Model 1** | | **Model 2** | | **Model 3** | | **Model 4** | |
| --- | --- | --- | --- | --- | --- | --- | --- | --- |
|  | **Difference (95% CI)** | **p-value** | **Difference (95% CI)** | **p-value** | **Difference (95% CI)** | **p-value** | **Difference (95% CI)** | **p-value** |
| **Arteriolar width** |  |  |  |  |  |  |  |  |
| *per SD in FI* | 0.19 (0.13,0.25) | 1.67E^-09^ | 0.12 (0.06,0.18) | 1.72E^-04^ | 0.10 (0.03,0.16) | 4.12E^-03^ | 0.12 (0.05,0.20) | 1.62E^-03^ |
| *per decade in age* | -0.84 (-0.92,-0.77) | 8.99E^-110^ | -0.63 (-0.70,-0.55) | 2.57E^-58^ | -0.08 (-0.17,0.01) | 0.07 | -0.05 (-0.15,0.06) | 0.38 |
| *Sex (female)* | -0.31 (-0.42,-0.19) | 4.49E^-07^ | 1.39 (1.21,1.56) | 3.29E^-56^ | 0.86 (0.67,1.05) | 1.91E^-18^ | 0.87 (0.65,1.10) | 3.27E^-14^ |
| *per 10 cm in height* |  |  | 1.27 (1.17,1.36) | 1.50E^-151^ | 1.17 (1.07,1.28) | 3.07E^-110^ | 1.23 (1.11,1.35) | 4.47E^-89^ |
| *BMI* |  |  |  |  | -0.02 (-0.04,-0.01) | 6.63E^-03^ | -0.01 (-0.03,0.00) | 0.12 |
| *HbA1c mmol/mol* |  |  |  |  | 0.04 (0.02,0.05) | 3.66E^-10^ | 0.03 (0.02,0.05) | 1.55E^-05^ |
| *per 10 mmHg in SBP* |  |  |  |  | -0.85 (-0.89,-0.81) | <10^-323^ | -0.92 (-0.97,-0.87) | <10^-323^ |
| *cholesterol mmol/L* |  |  |  |  | -0.09 (-0.15,-0.03) | 4.43E^-03^ | -0.05 (-0.13,0.02) | 0.16 |
| *triglycerides mmol/L* |  |  |  |  | 0.08 (0.00,0.15) | 0.04 | 0.07 (-0.02,0.16) | 0.12 |
| **Venular width** |  |  |  |  |  |  |  |  |
| *per SD in FI* | 0.29 (0.19,0.39) | 2.33E^-08^ | 0.30 (0.20,0.41) | 6.36E^-09^ | 0.32 (0.20,0.43) | 4.48E^-08^ | 0.32 (0.19,0.46) | 2.07E^-06^ |
| *per decade in age* | 0.59 (0.46,0.71) | 9.92E^-21^ | 0.72 (0.59,0.85) | 1.06E^-28^ | 0.80 (0.65,0.95) | 2.17E^-25^ | 0.80 (0.62,0.97) | 1.51E^-18^ |
| *Sex (female)* | -0.78 (-0.98,-0.59) | 7.54E^-15^ | 0.06 (-0.23,0.35) | 0.68 | 0.15 (-0.17,0.48) | 0.36 | 0.32 (-0.07,0.71) | 0.10 |
| *per 10 cm in height* |  |  | 0.58 (0.42,0.73) | 8.09E^-13^ | 0.61 (0.43,0.78) | 1.39E^-11^ | 0.63 (0.43,0.84) | 1.92E^-09^ |
| *BMI* |  |  |  |  | 0.11 (0.08,0.13) | 5.01E^-16^ | 0.11 (0.08,0.14) | 1.01E^-11^ |
| *HbA1c mmol/mol* |  |  |  |  | 0.03 (0.01,0.05) | 8.67E^-04^ | 0.04 (0.01,0.07) | 2.64E^-03^ |
| *per 10 mmHg in SBP* |  |  |  |  | -0.29 (-0.35,-0.22) | 7.47E^-18^ | -0.28 (-0.37,-0.20) | 1.24E^-11^ |
| *cholesterol mmol/L* |  |  |  |  | 0.01 (-0.09,0.11) | 0.82 | -0.05 (-0.18,0.08) | 0.44 |
| *triglycerides mmol/L* |  |  |  |  | 0.35 (0.22,0.48) | 8.75E^-08^ | 0.40 (0.25,0.56) | 3.49E^-07^ |
| **Arteriolar tortuosity**† |  |  |  |  |  |  |  |  |
| *per SD in FI* | 0.44 (0.04,0.84) | 0.03 | 0.52 (0.12,0.92) | 0.01 | 0.64 (0.19,1.09) | 0.01 | 0.78 (0.25,1.31) | 3.89E^-03^ |
| *per decade in age* | 2.15 (1.65,2.64) | 8.31E^-18^ | 2.19 (1.68,2.70) | 2.72E^-17^ | 1.02 (0.42,1.63) | 8.81E^-04^ | 0.69 (-0.01,1.40) | 0.05 |
| *Sex (female)* | 3.92 (3.11,4.72) | 2.40E^-22^ | 3.52 (2.35,4.69) | 2.02E^-09^ | 4.63 (3.28,6.00) | 9.80E^-12^ | 4.78 (3.19,6.40) | 2.3E-09 |
| *per 10 cm in height* |  |  | -0.33 (-0.95,0.29) | 0.29 | -0.23 (-0.92,0.47) | 0.52 | -0.33 (-1.14,0.49) | 0.43 |
| *BMI* |  |  |  |  | -0.15 (-0.25,-0.05) | 3.55E^-03^ | -0.31 (-0.43,-0.18) | 2.34E^-06^ |
| *HbA1c mmol/mol* |  |  |  |  | 0.13 (0.05,0.20) | 9.02E^-04^ | 0.09 (-0.01,0.19) | 0.09 |
| *per 10 mmHg in SBP* |  |  |  |  | 1.33 (1.06,1.59) | 3.72E^-23^ | 1.19 (0.86,1.52) | 9.35E^-13^ |
| *cholesterol mmol/L* |  |  |  |  | -0.04 (-0.45,0.37) | 0.85 | 0.24 (-0.26,0.75) | 0.35 |
| *triglycerides mmol/L* |  |  |  |  | 0.62 (0.11,1.13) | 0.02 | 0.72 (0.10,1.34) | 0.02 |
| **Venular tortuosity**† |  |  |  |  |  |  |  |  |
| *per SD in G4* | -0.68 (-0.92,-0.44) | 1.83E^-08^ | -0.59 (-0.83,-0.35) | 1.44E^-06^ | -0.33 (-0.59,-0.06) | 0.02 | -0.26 (-0.57,0.05) | 0.11 |
| *per decade in age* | 2.42 (2.12,2.72) | 2.23E^-58^ | 2.50 (2.19,2.80) | 4.04E^-58^ | 2.02 (1.65,2.38) | 5.08E^-28^ | 1.85 (1.43,2.27) | 3.99E^-18^ |
| *Sex (female)* | 1.27 (0.80,1.74) | 1.04E^-07^ | 1.20 (0.52,1.89) | 5.70E^-04^ | 1.98 (1.20,2.78) | 7.09E^-07^ | 1.75 (0.84,2.68) | 1.69E^-04^ |
| *per 10 cm in height* |  |  | -0.09 (-0.46,0.28) | 0.64 | 0.14 (-0.28,0.56) | 0.51 | 0.17 (-0.31,0.66) | 0.49 |
| *BMI* |  |  |  |  | 0.42 (0.36,0.48) | 1.19E^-41^ | 0.28 (0.21,0.36) | 1.71E^-13^ |
| *HbA1c mmol/mol* |  |  |  |  | 0.13 (0.09,0.18) | 4.46E^-09^ | 0.10 (0.04,0.16) | 1.68E^-03^ |
| *per 10 mmHg in SBP* |  |  |  |  | 0.48 (0.32,0.63) | 1.80E^-09^ | 0.41 (0.22,0.61) | 2.71E^-05^ |
| *cholesterol mmol/L* |  |  |  |  | -0.43 (-0.67,-0.19) | 4.91E^-04^ | -0.11 (-0.40,0.19) | 0.48 |
| *triglycerides mmol/L* |  |  |  |  | -0.46 (-0.76,-0.16) | 2.97E^-03^ | -0.54 (-0.90,-0.18) | 3.33E^-03^ |
| **Arteriolar area** |  |  |  |  |  |  |  |  |
| *per SD in FI* | 0.77 (0.17,1.38)* | 0.01 | 0.44 (-0.17,1.05)* | 0.16 | -0.10 (-0.78,0.57)* | 0.76 | -0.29 (-1.10,0.51)* | 0.47 |
| *per decade in age* | -0.36 (-0.37,-0.36) | <10^-323^ | -0.36 (-0.36,-0.35) | <10^-323^ | -0.32 (-0.33,-0.31) | <10^-323^ | -0.32 (-0.33,-0.31) | <10^-323^ |
| *Sex (female)* | -0.10 (-1.28,1.07) * | 0.86 | 0.08 (0.06,0.09) | 9.59E^-18^ | 0.02 (0.00,0.04) | 0.02 | 0.02 (-0.01,0.04) | 0.16 |
| *per 10 cm in height* |  |  | 0.06 (0.05,0.07) | 2.83E^-32^ | 0.05 (0.04,0.06) | 4.81E^-19^ | 0.05 (0.04,0.06) | 2.89E^-14^ |
| *BMI* |  |  |  |  | -0.01 (-0.01,-0.01) | 1.95E^-33^ | -0.01 (-0.01,-0.01) | 2.94E^-19^ |
| *HbA1c mmol/mol* |  |  |  |  | 0.00(-0.11,0.12)* | 0.95 | 0.08 (-0.08,0.23)* | 0.34 |
| *per 10 mmHg in SBP* |  |  |  |  | -0.06 (-0.06,-0.05) | 2.58E^-173^ | -0.06 (-0.06,-0.05) | 4.36E^-117^ |
| *cholesterol mmol/L* |  |  |  |  | 0.30 (-0.32,0.91)* | 0.34 | -0.07 (-0.84,0.70)* | 0.85 |
| *triglycerides mmol/L* |  |  |  |  | -0.36 (-1.12,0.40)* | 0.35 | 0.25 (-0.68,1.19)* | 0.60 |
| **Venular area** |  |  |  |  |  |  |  |  |
| *per SD in FI* | 0.05 (0.04,0.05) | 1.34E^-42^ | 0.04 (0.03,0.05) | 4.5E^-32^ | 0.04 (0.03,0.05) | 5.24E^-27^ | 0.05 (0.04,0.06) | 1.25E^-24^ |
| *per decade in age* | -0.07 (-0.08,-0.07) | 2.22E^-69^ | -0.06 (-0.07,-0.05) | 4.40E^-43^ | -0.05 (-0.06,-0.04) | 1.05E^-19^ | -0.05 (-0.06,-0.04) | 3.91E^-16^ |
| *Sex (female)* | -0.21 (-0.22,-0.20) | 1.11E^-214^ | -0.09 (-0.10,-0.07) | 1.65E^-18^ | -0.08 (-0.10,-0.06) | 1.80E^-13^ | -0.08 (-0.10,-0.05) | 1.04E^-08^ |
| *per 10 cm in height* |  |  | 0.09 (0.08,0.10) | 5.49E^-67^ | 0.10 (0.08,0.11) | 5.22E^-57^ | 0.09 (0.08,0.11) | 2.86E^-40^ |
| *BMI* |  |  |  |  | 0.01 (0.01,0.01) | 9.27E^-26^ | 0.01 (0.01,0.01) | 4.58E^-13^ |
| *HbA1c mmol/mol* |  |  |  |  | 0.09 (-0.04,0.21)* | 0.18 | 0.08 (-0.10,0.25)* | 0.38 |
| *per 10 mmHg in SBP* |  |  |  |  | -0.02(-0.03,-0.02) | 2.39E^-26^ | -0.02 (-0.03,-0.02) | 4.9E^-16^ |
| *cholesterol mmol/L* |  |  |  |  | 0.01 (0.00,0.02) | 1.98E^-03^ | 0.01 (0.00,0.02) | 0.01 |
| *triglycerides mmol/L* |  |  |  |  | 0.01 (0.00,0.02) | 0.04 | 0.01 (0.00,0.02) | 0.03 |
| **1/ (Arteriolar SD(µm))** * |  |  |  |  |  |  |  |  |
| *per SD in FI* | -0.07 (-0.09,-0.05) | 1.27E^-11^ | -0.07 (-0.09,-0.05) | 1.34E^-11^ | -0.08 (-0.10,-0.06) | 1.1E^-13^ | -0.08 (-0.11,-0.05)* | 2.4E^-09^ |
| *per decade in age* | -0.92 (-0.94,-0.89) | <10^-323^ | -0.92 (-0.95,-0.90) | <10^-323^ | -0.84 (-0.87,-0.81) | <10^-323^ | -0.86 (-0.89,-0.83) | <10^-323^ |
| *Sex (female)* | 0.08 (0.04,0.12) | 2.81E^-05^ | 0.04 (-0.02,0.09) | 0.17 | -0.05 (-0.11,0.01) | 0.13 | -0.07 (-0.15,0.00) | 0.05 |
| *per 10 cm in height* |  |  | -0.03 (-0.06,0.00) | 0.07 | -0.05 (-0.08,-0.01) | 8.52E^-03^ | -0.04 (-0.08,0.00) | 0.07 |
| *BMI* |  |  |  |  | -0.01 (-0.01,0.00) | 8.20E^-04^ | -0.01 (-0.02,-0.01) | 1.40E^-04^ |
| *HbA1c mmol/mol* |  |  |  |  | 0.00 (-0.01,0.00) | 0.21 | 0.00 (-0.01,0.00) | 0.18 |
| *per 10 mmHg in SBP* |  |  |  |  | -0.11 (-0.12,-0.10) | 5.49E^-63^ | -0.11 (-0.13,-0.10) | 3.47E^-42^ |
| *cholesterol mmol/L* |  |  |  |  | -0.02 (-0.04,0.00) | 0.10 | 0.00 (-0.03,0.02) | 0.90 |
| *triglycerides mmol/L* |  |  |  |  | 0.00 (-0.03,0.02) | 0.93 | -0.01 (-0.04,0.02) | 0.62 |
| **1/ (Venular SD(µm))** * |  |  |  |  |  |  |  |  |
| *per SD in FI* | -0.07 (-0.09,-0.05) | 1.52E^-09^ | -0.07 (-0.09,-0.05) | 2.10E^-09^ | -0.07 (-0.10,-0.05) | 5.24E^-09^ | -0.07 (-0.10,-0.04) | 1.27E^-06^ |
| *per decade in age* | -1.02 (-1.04,-0.99) | <10^-323^ | -1.03 (-1.06,-1.01) | <10^-323^ | -1.03 (-1.06,-0.99) | <10^-323^ | -1.03 (-1.07,-0.99) | <10^-323^ |
| *Sex (female)* | 0.00 (-0.04,0.04) | 0.94 | -0.09 (-0.16,-0.03) | 3.32E^-03^ | -0.13 (-0.20,-0.06) | 4.87E^-04^ | -0.18 (-0.27,-0.09) | 5.98E^-05^ |
| *per 10 cm in height* |  |  | -0.07 (-0.10,-0.03) | 1.10E^-04^ | -0.08 (-0.12,-0.04) | 5.26E^-05^ | -0.10 (-0.14,-0.05) | 5.63E^-05^ |
| *BMI* |  |  |  |  | -0.02 (-0.02,-0.01) | 2.08E^-08^ | -0.02 (-0.03,-0.01) | 1.42E^-06^ |
| *HbA1c mmol/mol* |  |  |  |  | -0.01 (-0.01,0.00) | 1.52E^-04^ | -0.01 (-0.01,0.00) | 0.02 |
| *per 10 mmHg in SBP* |  |  |  |  | 0.02 (0.00,0.03) | 0.03 | 0.02 (0.00,0.04) | 0.03 |
| *cholesterol mmol/L* |  |  |  |  | 0.01 (-0.01,0.03) | 0.49 | 0.00 (-0.03,0.03) | 0.91 |
| *triglycerides mmol/L* |  |  |  |  | 0.00 (-0.03,0.03) | 0.88 | 0.00 (-0.04,0.03) | 0.98 |

† - Coefficients as % change for log transformed target variables. * - Coefficients multiplied by 100. Model 1 - adjusted for age, sex, ethnicity and UK Biobank centre. Model 2 - Model 1 adjustment, with adjustment for smoking, Townsend deprivation index and height. Model 3 – Model 2 adjustment, with adjustment for BMI, HbA1c, systolic BP, total cholesterol and triacylglycerols. Model 4 – Same as Model 3 but excluding persons with self-reported history of heart attack, stroke, hypertension or on medication for hypertension.

**eTable 8: Difference in retinal vasculometry characteristics between participants that completed the prospective memory (PM) test**

**and other covariates from multivariable regression models with different levels of adjustment.**

|  | **Model 1** | | **Model 2** | | **Model 3** | | **Model 4** | |
| --- | --- | --- | --- | --- | --- | --- | --- | --- |
|  | **Difference (95% CI)** | **p-value** | **Difference (95% CI)** | **p-value** | **Difference (95% CI)** | **p-value** | **Difference (95% CI)** | **p-value** |
| **Arteriolar width** |  |  |  |  |  |  |  |  |
| *PM (answered correctly)* | 0.15 (-0.01,0.31) | 6.95E^-02^ | 0.06 (-0.11,0.22) | 0.49 | 0.08  (-0.09,0.25) | 0.36 | 0.04  (-0.16,0.25) | 0.70 |
| *per decade in age* | -0.86 (-0.93,-0.78) | 1.52^E-112^ | -0.63  (-0.71,-0.56) | 1.82E^-59^ | -0.08  (-0.17,0.00) | 0.06 | -0.05  (-0.15,0.05) | 0.33 |
| *Sex (female)* | -0.33 (-0.44,-0.21) | 7.35E^-08^ | 1.40  (1.23,1.57) | 2.82E^-57^ | 0.87 (0.68,1.06) | 1.02E^-18^ | 0.88  (0.66,1.11) | 1.48E^-14^ |
| *per 10 cm in height* |  |  | 1.29  (1.19,1.38) | 3.62E^-158^ | 1.19  (1.09,1.29) | 3.35E^-114^ | 1.25  (1.13,1.37) | 4.23E^-93^ |
| *BMI* |  |  |  |  | -0.02  (-0.04,-0.01) | 5.74E^-03^ | -0.02  (-0.03,0.00) | 0.11 |
| *HbA1c mmol/mol* |  |  |  |  | 0.03  (0.02,0.05) | 4.52E^-10^ | 0.03  (0.02,0.05) | 1.88E^-05^ |
| *per 10 mmHg in SBP* |  |  |  |  | -0.85  (-0.89,-0.81) | <10^-323^ | -0.92  (-0.97,-0.87) | <10^-323^ |
| *cholesterol mmol/L* |  |  |  |  | -0.09  (-0.15,-0.03) | 0.01 | -0.05  (-0.13,0.02) | 0.17 |
| *triglycerides mmol/L* |  |  |  |  | 0.08  (0.00,0.15) | 0.04 | 0.07  (-0.02,0.16) | 0.11 |
| **Venular width** |  |  |  |  |  |  |  |  |
| *PM (answered correctly)* | 0.64 (0.37,0.91) | 4.02E^-06^ | 0.66  (0.38,0.93) | 2.29E^-06^ | 0.62  (0.33,0.91) | 3.73E^-05^ | 0.58  (0.23,0.94) | 1.21E^-03^ |
| *per decade in age* | 0.59 (0.46,0.71) | 1.14E^-20^ | 0.73  (0.60,0.85) | 7.69E-^29^ | 0.81  (0.66,0.96) | 1.14E^-25^ | 0.80  (0.63,0.98) | 9.05E^-19^ |
| *Sex (female)* | -0.81 (-1.01,-0.61) | 9.10E^-16^ | 0.08  (-0.21,0.37) | 0.59 | 0.17  (-0.16,0.50) | 0.32 | 0.34  (-0.05,0.73) | 0.09 |
| *per 10 cm in height* |  |  | 0.61  (0.46,0.77) | 1.95E^-14^ | 0.64  (0.47,0.82) | 5.72E^-13^ | 0.67  (0.47,0.88) | 1.43E^-10^ |
| *BMI* |  |  |  |  | 0.10  (0.08,0.13) | 1.12E^-15^ | 0.11  (0.08,0.14) | 1.59E^-11^ |
| *HbA1c mmol/mol* |  |  |  |  | 0.03  (0.01,0.05) | 9.03E^-04^ | 0.04  (0.01,0.07) | 2.71E^-03^ |
| *per 10 mmHg in SBP* |  |  |  |  | -0.29  (-0.36,-0.23) | 1.48E^-18^ | -0.29  (-0.37,-0.21) | 4.16E^-12^ |
| *cholesterol mmol/L* |  |  |  |  | 0.01  (-0.09,0.12) | 0.78 | -0.05  (-0.18,0.08) | 0.46 |
| *triglycerides mmol/L* |  |  |  |  | 0.35  (0.22,0.48) | 7.48E^-08^ | 0.41  (0.25,0.56) | 2.87E^-07^ |
| **Arteriolar tortuosity**† |  |  |  |  |  |  |  |  |
| *PM (answered correctly)* | 1.07  (0.00,2.15) | 0.05 | 1.19  (0.11,2.27) | 0.03 | 1.28  (0.10,2.47) | 0.03 | 1.70  (0.29,3.13) | 0.02 |
| *per decade in age* | 2.15  (1.66,2.65) | 8.13E^-18^ | 2.20  (1.68,2.71) | 2.42E^-17^ | 1.04  (0.43,1.65) | 7.62E^-04^ | 0.72  (0.02,1.44) | 0.04 |
| *Sex (female)* | 3.88  (3.08,4.68) | 5.20E^-22^ | 3.55  (2.39,4.72) | 1.42E^-09^ | 4.65  (3.30,6.03) | 7.50E^-12^ | 4.82  (3.23,6.44) | 1.71E^-09^ |
| *per 10 cm in height* |  |  | -0.28  (-0.89,0.34) | 0.38 | -0.15  (-0.84,0.54) | 0.66 | -0.25  (-1.06,0.57) | 0.55 |
| *BMI* |  |  |  |  | -0.15  (-0.25,-0.05) | 3.03E^-03^ | -0.31  (-0.44,-0.18) | 1.92E^-06^ |
| *HbA1c mmol/mol* |  |  |  |  | 0.13  (0.05,0.20) | 9.19E^-04^ | 0.09  (-0.01,0.19) | 0.09 |
| *per 10 mmHg in SBP* |  |  |  |  | 1.31  (1.05,1.58) | 9.05E^-23^ | 1.18  (0.85,1.51) | 1.83E^-12^ |
| *cholesterol mmol/L* |  |  |  |  | -0.03  (-0.44,0.38) | 0.88 | 0.25  (-0.26,0.75) | 0.34 |
| *triglycerides mmol/L* |  |  |  |  | 0.62  (0.12,1.13) | 0.02 | 0.73  (0.11,1.35) | 0.02 |
| **Venular tortuosity**† |  |  |  |  |  |  |  |  |
| *PM (answered correctly)* | -1.26  (-1.89,-0.63) | 9.67E^-05^ | -1.10  (-1.73,-0.46) | 7.47E^-04^ | -0.94  (-1.62,-0.24) | 0.01 | -0.79  (-1.60,0.03) | 0.06 |
| *per decade in age* | 2.43  (2.13,2.73) | 1.13E^-58^ | 2.50  (2.19,2.81) | 7.14E^-58^ | 2.00  (1.63,2.36) | 2.84E^-27^ | 1.83  (1.41,2.25) | 1.22E^-17^ |
| *Sex (female)* | 1.33  (0.86,1.81) | 2.23E^-08^ | 1.16  (0.47,1.84) | 8.90E^-04^ | 1.98  (1.19,2.77) | 7.56E^-07^ | 1.75  (0.83,2.67) | 1.78E^-04^ |
| *per 10 cm in height* |  |  | -0.17  (-0.54,0.20) | 0.38 | 0.11  (-0.30,0.53) | 0.60 | 0.15  (-0.33,0.63) | 0.54 |
| *BMI* |  |  |  |  | 0.42  (0.36,0.48) | 6.19E^-42^ | 0.29  (0.21,0.36) | 1.41E^-13^ |
| *HbA1c mmol/mol* |  |  |  |  | 0.13  (0.09,0.18) | 4.84E^-09^ | 0.10  (0.04,0.16) | 1.77E^-03^ |
| *per 10 mmHg in SBP* |  |  |  |  | 0.49  (0.33,0.64) | 1.03E^-09^ | 0.42  (0.23,0.61) | 2.07E^-05^ |
| *cholesterol mmol/L* |  |  |  |  | -0.43  (-0.68,-0.19) | 4.67E^-04^ | -0.11  (-0.40,0.19) | 0.47 |
| *triglycerides mmol/L* |  |  |  |  | -0.46  (-0.76,-0.16) | 2.82E^-03^ | -0.54  (-0.90,-0.18) | 3.18E^-03^ |
| **Arteriolar area** |  |  |  |  |  |  |  |  |
| *PM (answered correctly)* | 0.02  (0.00,0.04) | 0.01 | 0.02  (0.00,0.03) | 0.05 | 0.02  (0.00,0.04) | 0.03 | 1.81  (-0.32,3.94)* | 0.10 |
| *per decade in age* | -0.36  (-0.37,-0.36) | <10^-323^ | -0.35  (-0.36,-0.35) | <10^-323^ | -0.32  (-0.32,-0.31) | <10^-323^ | -0.32  (-0.33,-0.31) | <10^-323^ |
| *Sex (female)* | -0.17  (-1.35,1.01) * | 0.78 | 0.08  (0.06,0.09) | 8.34E^-18^ | 0.02  (0.00,0.04) | 0.02 | 0.02  (-0.01,0.04) | 0.18 |
| *per 10 cm in height* |  |  | 0.06  (0.05,0.07) | 5.55E^-33^ | 0.05  (0.04,0.06) | 1.29E^-18^ | 0.05  (0.03,0.06) | 8.46E^-14^ |
| *BMI* |  |  |  |  | -0.01  (-0.01,-0.01) | 1.86E^-33^ | -0.01  (-0.01,-0.01) | 2.90E^-19^ |
| *HbA1c mmol/mol* |  |  |  |  | 0.01  (-0.10,0.12)* | 0.90 | 0.08  (-0.08,0.24)* | 0.32 |
| *per 10 mmHg in SBP* |  |  |  |  | -0.06  (-0.06,-0.05) | 1.40E^-173^ | -0.06  (-0.06,-0.05) | 4.35E^-117^ |
| *cholesterol mmol/L* |  |  |  |  | 0.28  (-0.33,0.89)* | 0.37 | -0.09  (-0.86,0.68)* | 0.82 |
| *triglycerides mmol/L* |  |  |  |  | -0.36  (-1.12,0.40)* | 0.35 | 0.25  (-0.68,1.19)* | 0.60 |
| **Venular area** |  |  |  |  |  |  |  |  |
| *PM (answered correctly)* | 0.05  (0.03,0.06) | 5.07E^-07^ | 0.04  (0.02,0.06) | 3.51E^-05^ | 0.04  (0.02,0.06) | 1.06E^-05^ | 0.06 (0.03,0.08) | 3.52E^-06^ |
| *per decade in age* | -0.08  (-0.08,-0.07) | 4.31E^-74^ | -0.06  (-0.07,-0.05) | 1.70E^-45^ | -0.05  (-0.06,-0.04) | 3.05E^-20^ | -0.05 (-0.06,-0.04) | 4.21E^-16^ |
| *Sex (female)* | -0.21  (-0.23,-0.20) | 6.82E^-225^ | -0.08  (-0.10,-0.06) | 5.81E^-17^ | -0.08  (-0.10,-0.06) | 1.07E^-12^ | -0.07 (-0.10,-0.05) | 4.49E^-08^ |
| *per 10 cm in height* |  |  | 0.10  (0.09,0.11) | 3.89E^-77^ | 0.10  (0.09,0.11) | 8.11E^-65^ | 0.10 (0.09,0.12) | 2.56E^-46^ |
| *BMI* |  |  |  |  | 0.01  (0.01,0.01) | 6.82E^-25^ | 0.01 (0.01,0.01) | 1.22E^-12^ |
| *HbA1c mmol/mol* |  |  |  |  | 0.08 (-0.05,0.20)* | 0.21 | 0.07 (-0.10,0.25)* | 0.41 |
| *per 10 mmHg in SBP* |  |  |  |  | -0.02  (-0.03,-0.02) | 7.60E^-28^ | -0.02 (-0.03,-0.02) | 3.62E^-17^ |
| *cholesterol mmol/L* |  |  |  |  | 0.01  (0.00,0.02) | 1.04E^-03^ | 0.01 (0.00,0.02) | 6.45E^-03^ |
| *triglycerides mmol/L* |  |  |  |  | 0.01  (0.00,0.02) | 0.03 | 0.01 (0.00,0.02) | 0.02 |
| **1/ (Arteriolar SD(µm))*** |  |  |  |  |  |  |  |  |
| *PM (answered correctly)* | -0.06 (-0.12,-0.01) | 0.01 | -0.06 (-0.12,-0.01) | 0.02 | -0.06 (-0.11,0.00) | 0.05 | -0.05 (-0.11,0.02) | 0.20 |
| *per decade in age* | -0.91 (-0.94,-0.89) | <10^-323^ | -0.92 (-0.94,-0.90) | <10^-323^ | -0.84 (-0.87,-0.81) | <10^-323^ | -0.86 (-0.89,-0.82) | <10^-323^ |
| *Sex (female)* | 0.09 (0.05,0.12) | 5.21E^-06^ | 0.03 (-0.02,0.09) | 0.26 | -0.06 (-0.12,0.01) | 0.09 | -0.08 (-0.16,-0.01) | 0.03 |
| *per 10 cm in height* |  |  | -0.04 (-0.07,-0.01) | 0.01 | -0.06 (-0.09,-0.02) | 6.50E^-04^ | -0.05 (-0.09,-0.01) | 0.01 |
| *BMI* |  |  |  |  | -0.01 (-0.01,0.00) | 1.27E^-03^ | -0.01  (-0.02,-0.01) | 1.84E^-04^ |
| *HbA1c mmol/mol* |  |  |  |  | 0.00 (-0.01,0.00) | 0.25 | 0.00  (-0.01,0.00) | 0.20 |
| *per 10 mmHg in SBP* |  |  |  |  | -0.11 (-0.12,-0.09) | 2.10E^-61^ | -0.11  (-0.13,-0.09) | 3.90E^-41^ |
| *cholesterol mmol/L* |  |  |  |  | -0.02 (-0.04,0.00) | 0.07 | 0.00  (-0.03,0.02) | 0.83 |
| *triglycerides mmol/L* |  |  |  |  | 0.00 (-0.03,0.02) | 0.90 | -0.01  (-0.04,0.02) | 0.59 |
| **1/ (Venular SD(µm))*** |  |  |  |  |  |  |  |  |
| *PM (answered correctly)* | -0.03  (-0.09,0.03) | 0.30 | -0.03  (-0.09,0.03) | 0.30 | -0.03  (-0.09,0.04) | 0.44 | -0.03  (-0.11,0.05) | 0.47 |
| *per decade in age* | -1.01  (-1.04,-0.98) | <10^-323^ | -1.03  (-1.06,-1.00) | <10^-323^ | -1.02  (-1.06,-0.99) | <10^-323^ | -1.02  (-1.06,-0.98) | <10^-323^ |
| *Sex (female)* | 0.01  (-0.03,0.05) | 0.67 | -0.10  (-0.17,-0.04) | 1.51E^-03^ | -0.14  (-0.21,-0.06) | 2.58E^-04^ | -0.19  (-0.27,-0.10) | 3.12E^-05^ |
| *per 10 cm in height* |  |  | -0.08  (-0.11,-0.05) | 5.04E^-06^ | -0.09  (-0.13,-0.05) | 2.50E^-06^ | -0.11  (-0.16,-0.06) | 4.60E^-06^ |
| *BMI* |  |  |  |  | -0.02  (-0.02,-0.01) | 3.58E^-08^ | -0.02  (-0.03,-0.01) | 1.85E^-06^ |
| *HbA1c mmol/mol* |  |  |  |  | -0.01  (-0.01,0.00) | 2.17E^-04^ | -0.01  (-0.01,0.00) | 0.02 |
| *per 10 mmHg in SBP* |  |  |  |  | 0.02  (0.00,0.03) | 0.02 | 0.02  (0.00,0.04) | 0.02 |
| *cholesterol mmol/L* |  |  |  |  | 0.01  (-0.02,0.03) | 0.57 | 0.00  (-0.03,0.03) | 0.85 |
| *triglycerides mmol/L* |  |  |  |  | 0.00  (-0.03,0.03) | 0.87 | 0.00  (-0.04,0.03) | 0.95 |

† - Coefficients as % change for log transformed target variables. * - Coefficients multiplied by 100. Model 1 - adjusted for age, sex, ethnicity, and UK Biobank centre. Model 2 - Model 1 adjustment, with adjustment for smoking, Townsend deprivation index and height. Model 3 – Model 2 adjustment, with adjustment for BMI, HbA1c, systolic BP, total cholesterol and triacylglycerols. Model 4 – Same as Model 3 but excluding persons with self-reported history of heart attack, stroke, hypertension or on medication for hypertension.

**eTable 9: Difference in retinal vasculometry characteristics per SD increase in log transformed pairs matching score (PS) and other covariates from multivariable regression models with different levels of adjustment.**

|  | **Model 1** | | **Model 2** | | **Model 3** | | **Model 4** | |
| --- | --- | --- | --- | --- | --- | --- | --- | --- |
|  | **Difference (95% CI)** | **p-value** | **Difference (95% CI)** | **p-value** | **Difference (95% CI)** | **p-value** | **Difference (95% CI)** | **p-value** |
| **Arteriolar width** |  |  |  |  |  |  |  |  |
| *per SD in PS* | -0.10  (-0.16,-0.04) | 9.80E^-04^ | -0.08  (-0.14,-0.02) | 0.01 | -0.08  (-0.15,-0.02) | 0.01 | -0.10(-0.18,-0.03) | 0.01 |
| *per decade in age* | -0.84  (-0.92,-0.77) | 6.55E^-108^ | -0.62  (-0.70,-0.54) | 1.68E^-56^ | -0.07  (-0.16,0.02) | 0.11 | -0.03(-0.14,0.07) | 0.52 |
| *Sex (female)* | -0.33  (-0.44,-0.21) | 7.20E^-08^ | 1.40  (1.22,1.57) | 3.78E^-57^ | 0.87  (0.67,1.06) | 1.19E^-18^ | 0.88(0.65,1.10) | 2.03E^-14^ |
| *per 10 cm in height* |  |  | 1.28  (1.19,1.38) | 5.27E^-158^ | 1.19  (1.08,1.29) | 3.50E^-114^ | 1.25(1.13,1.37) | 1.02E^-92^ |
| *BMI* |  |  |  |  | -0.02  (-0.04,-0.01) | 4.68E^-03^ | -0.02(-0.03,0.00) | 0.10 |
| *HbA1c mmol/mol* |  |  |  |  | 0.03  (0.02,0.05) | 4.97E^-10^ | 0.03(0.02,0.05) | 1.80E^-05^ |
| *per 10 mmHg in SBP* |  |  |  |  | -0.85  (-0.89,-0.81) | 0 | -0.92(-0.97,-0.87) | 0 |
| *cholesterol mmol/L* |  |  |  |  | -0.09  (-0.15,-0.03) | 0.01 | -0.05(-0.13,0.02) | 0.17 |
| *triglycerides mmol/L* |  |  |  |  | 0.08  (0.00,0.15) | 0.04 | 0.07(-0.02,0.16) | 0.12 |
| **Venular width** |  |  |  |  |  |  |  |  |
| *per SD in PS* | -0.24  (-0.34,-0.14) | 2.18E^-06^ | -0.23  (-0.33,-0.13) | 4.78E^-06^ | -0.21  (-0.32,-0.10) | 2.51E^-04^ | -0.20(-0.33,-0.07) | 2.27E^-03^ |
| *per decade in age* | 0.60  (0.48,0.73) | 1.81E^-21^ | 0.74  (0.61,0.87) | 1.67E^-29^ | 0.82  (0.67,0.97) | 6.03E^-26^ | 0.82(0.64,0.99) | 4.29E^-19^ |
| *Sex (female)* | -0.81  (-1.01,-0.62) | 6.48E^-16^ | 0.09  (-0.20,0.37) | 0.55 | 0.17  (-0.16,0.50) | 0.30 | 0.34(-0.04,0.73) | 0.08 |
| *per 10 cm in height* |  |  | 0.62  (0.47,0.78) | 7.30E^-15^ | 0.65  (0.48,0.83) | 2.29E^-13^ | 0.68(0.48,0.89) | 7.92E^-11^ |
| *BMI* |  |  |  |  | 0.10  (0.08,0.13) | 2.36E^-15^ | 0.11(0.08,0.14) | 2.31E^-11^ |
| *HbA1c mmol/mol* |  |  |  |  | 0.03  (0.01,0.05) | 1.21E^-03^ | 0.04(0.01,0.07) | 3.18E^-03^ |
| *per 10 mmHg in SBP* |  |  |  |  | -0.29  (-0.36,-0.23) | 2.10E^-18^ | -0.29(-0.37,-0.21) | 4.84E^-12^ |
| *cholesterol mmol/L* |  |  |  |  | 0.02  (-0.09,0.12) | 0.75 | -0.05(-0.17,0.08) | 0.47 |
| *triglycerides mmol/L* |  |  |  |  | 0.35  (0.22,0.48) | 8.40E^-08^ | 0.40(0.25,0.56) | 3.19E^-07^ |
| **Arteriolar tortuosity**† |  |  |  |  |  |  |  |  |
| *per SD in PS* | -0.22  (-0.60,0.17) | 0.28 | -0.23  (-0.62,0.16) | 0.25 | -0.31  (-0.74,0.13) | 0.17 | -0.37 (-0.88,0.13) | 0.15 |
| *per decade in age* | 2.14  (1.64,2.64) | 1.86E^-17^ | 2.18  (1.67,2.70) | 5.60E^-17^ | 1.03  (0.42,1.64) | 8.49E^-04^ | 0.72  (0.01,1.44) | 0.05 |
| *Sex (female)* | 3.87  (3.07,4.67) | 6.63E^-22^ | 3.57  (2.41,4.75) | 1.11E^-09^ | 4.67  (3.32,6.05) | 6.14E^-12^ | 4.84  (3.25,6.46) | 1.46E^-09^ |
| *per 10 cm in height* |  |  | -0.25  (-0.86,0.37) | 0.43 | -0.13  (-0.82,0.57) | 0.72 | -0.21  (-1.02,0.61) | 0.61 |
| *BMI* |  |  |  |  | -0.15  (-0.26,-0.05) | 2.74E^-03^ | -0.31  (-0.44,-0.18) | 1.74E^-06^ |
| *HbA1c mmol/mol* |  |  |  |  | 0.12  (0.05,0.20) | 1.07E^-03^ | 0.09  (-0.02,0.19) | 0.09 |
| *per 10 mmHg in SBP* |  |  |  |  | 1.32  (1.05,1.58) | 7.43E^-23^ | 1.18  (0.85,1.51) | 1.65E^-12^ |
| *cholesterol mmol/L* |  |  |  |  | -0.03  (-0.43,0.38) | 0.90 | 0.25  (-0.25,0.76) | 0.33 |
| *triglycerides mmol/L* |  |  |  |  | 0.62  (0.11,1.13) | 0.02 | 0.72  (0.11,1.34) | 0.02 |
| **Venular tortuosity**† |  |  |  |  |  |  |  |  |
| *per SD in PS* | 0.06  (-0.17,0.30) | 0.59 | 0.04  (-0.19,0.28) | 0.74 | 0.00  (-0.26,0.26) | 0.98 | -0.02  (-0.32,0.28) † | 0.89 |
| *per decade in age* | 2.48  (2.18,2.78) | 3.33E^-60^ | 2.54  (2.23,2.86) | 3.03E^-59^ | 2.04  (1.68,2.41) | 3.41E^-28^ | 1.87  (1.45,2.30) † | 3.58E^-18^ |
| *Sex (female)* | 1.35  (0.88,1.82) | 1.51E^-08^ | 1.13  (0.44,1.82) | 1.19E^-03^ | 1.95  (1.17,2.75) | 1.03E^-06^ | 1.73  (0.81,2.65) † | 2.12E^-04^ |
| *per 10 cm in height* |  |  | -0.20  (-0.57,0.17) | 0.29 | 0.08  (-0.33,0.49) | 0.70 | 0.12  (-0.36,0.60) † | 0.62 |
| *BMI* |  |  |  |  | 0.42  (0.36,0.48) | 7.56E^-42^ | 0.28  (0.21,0.36) † | 1.57E^-13^ |
| *HbA1c mmol/mol* |  |  |  |  | 0.13  (0.09,0.18) | 3.43E^-09^ | 0.10  (0.04,0.16) † | 1.53E^-03^ |
| *per 10 mmHg in SBP* |  |  |  |  | 0.48  (0.33,0.64) | 1.20E^-09^ | 0.42  (0.22,0.61) † | 2.20E^-05^ |
| *cholesterol mmol/L* |  |  |  |  | -0.44  (-0.68,-0.20) | 3.93E^-04^ | -0.11  (-0.41,0.18) † | 0.46 |
| *triglycerides mmol/L* |  |  |  |  | -0.46  (-0.76,-0.16) | 2.90E^-03^ | -0.54  (-0.90,-0.18) † | 3.20E^-03^ |
| **Arteriolar area** |  |  |  |  |  |  |  |  |
| *per SD in PS* | 0.12 (-0.47,0.72)* | 0.68 | 0.25 (-0.34,0.85)* | 0.40 | 0.12 (-0.53,0.78)* | 0.71 | -0.32  (-1.10,0.46)* | 0.42 |
| *per decade in age* | -0.37  (-0.37,-0.36) | <10^-323^ | -0.36  (-0.36,-0.35) | <10^-323^ | -0.32  (-0.33,-0.31) | <10^-323^ | -0.32  (-0.33,-0.31) | <10^-323^ |
| *Sex (female)* | -0.20  (-1.37,0.98) * | 0.74 | 0.08  (0.06,0.09) | 4.72E^-18^ | 0.02  (0.00,0.04) | 0.02 | 0.02  (-0.01,0.04) | 0.17 |
| *per 10 cm in height* |  |  | 0.06  (0.05,0.07) | 9.83E^-34^ | 0.05  (0.04,0.06) | 3.55E^-19^ | 0.05  (0.04,0.06) | 4.71E^-14^ |
| *BMI* |  |  |  |  | -0.01  (-0.01,-0.01) | 2.42E^-33^ | -0.01  (-0.01,-0.01) | 2.71E^-19^ |
| *HbA1c mmol/mol* |  |  |  |  | 0.00  (-0.11,0.12)* | 0.94 | 0.08  (-0.08,0.23)* | 0.34 |
| *per 10 mmHg in SBP* |  |  |  |  | -0.06  (-0.06,-0.05) | 2.43E^-173^ | -0.06  (-0.06,-0.05) | 5.63E^-117^ |
| *cholesterol mmol/L* |  |  |  |  | 0.29  (-0.32,0.91)* | 0.35 | -0.08(-0.85,0.69)* | 0.84 |
| *triglycerides mmol/L* |  |  |  |  | -0.36  (-1.12,0.40)* | 0.35 | 0.25  (-0.69,1.18)* | 0.60 |
| **Venular area** |  |  |  |  |  |  |  |  |
| *per SD in PS* | -0.01  (-0.01,0.00) | 0.01 | -0.01  (-0.01,0.00) | 0.06 | -0.01  (-0.01,0.00) | 0.10 | -0.01  (-0.01,0.00) | 0.24 |
| *per decade in age* | -0.08  (-0.09,-0.07) | 2.84E^-74^ | -0.06  (-0.07,-0.05) | 8.11E^-46^ | -0.05  (-0.06,-0.04) | 7.72E^-21^ | -0.05  (-0.06,-0.04) | 7.45E^-17^ |
| *Sex (female)* | -0.22  (-0.23,-0.20) | 7.65E^-226^ | -0.08  (-0.10,-0.06) | 1.11E^-16^ | -0.08  (-0.10,-0.06) | 1.87E^-12^ | -0.07  (-0.10,-0.05) | 7.19E^-08^ |
| *per 10 cm in height* |  |  | 0.10  (0.09,0.11) | 1.26E^-78^ | 0.10 (0.09,0.11) | 2.16E^-66^ | 0.10  (0.09,0.12) | 8.00E^-48^ |
| *BMI* |  |  |  |  | 0.01  (0.01,0.01) | 1.01E^-24^ | 0.01  (0.01,0.01) | 1.28E^-12^ |
| *HbA1c mmol/mol* |  |  |  |  | 0.00  (0.00,0.00) | 0.25 | 0.00  (0.00,0.00) | 0.47 |
| *per 10 mmHg in SBP* |  |  |  |  | -0.02  (-0.03,-0.02) | 1.21E^-27^ | -0.02  (-0.03,-0.02) | 4.78E^-17^ |
| *cholesterol mmol/L* |  |  |  |  | 0.01  (0.00,0.02) | 8.37E^-04^ | 0.01  (0.00,0.02) | 5.47E^-03^ |
| *triglycerides mmol/L* |  |  |  |  | 0.01  (0.00,0.02) | 0.03 | 0.01  (0.00,0.02) | 0.03 |
| **1/ (Arteriolar SD(µm))*** |  |  |  |  |  |  |  |  |
| *per SD in PS* | 0.02  (0.00,0.03) | 0.12 | 0.02  (0.00,0.03) | 0.12 | 0.01  (-0.01,0.03) | 0.37 | 0.01 (-0.01,0.04) | 0.36 |
| *per decade in age* | -0.91  (-0.94,-0.89) | <10^-323^ | -0.92  (-0.94,-0.90) | <10^-323^ | -0.84  (-0.87,-0.81) | <10^-323^ | -0.86 (-0.89,-0.82) | <10^-323^ |
| *Sex (female)* | 0.09  (0.05,0.13) | 4.51E^-06^ | 0.03  (-0.02,0.09) | 0.27 | -0.06  (-0.12,0.01) | 0.08 | -0.08 (-0.16,-0.01) | 0.03 |
| *per 10 cm in height* |  |  | -0.04  (-0.07,-0.01) | 0.01 | -0.06  (-0.09,-0.03) | 4.77E^-04^ | -0.05 (-0.09,-0.01) | 0.01 |
| *BMI* |  |  |  |  | -0.01  (-0.01,0.00) | 1.36E^-03^ | -0.01 (-0.02,-0.01) | 1.95E^-04^ |
| *HbA1c mmol/mol* |  |  |  |  | 0.00  (-0.01,0.00) | 0.27 | 0.00  (-0.01,0.00) | 0.21 |
| *per 10 mmHg in SBP* |  |  |  |  | -0.11  (-0.12,-0.09) | 1.56E^-61^ | -0.11  (-0.13,-0.09) | 3.47E^-41^ |
| *cholesterol mmol/L* |  |  |  |  | -0.02  (-0.04,0.00) | 0.07 | 0.00  (-0.03,0.02) | 0.82 |
| *triglycerides mmol/L* |  |  |  |  | 0.00  (-0.03,0.02) | 0.91 | -0.01  (-0.04,0.02) | 0.60 |
| **1/ (Venular SD(µm))*** |  |  |  |  |  |  |  |  |
| *per SD in PS* | 0.03  (0.01,0.05) | 0.01 | 0.03  (0.01,0.05) | 4.35E^-03^ | 0.03  (0.00,0.05) | 0.04 | 0.02  (-0.01,0.05) | 0.11 |
| *per decade in age* | -1.02  (-1.04,-0.99) | <10^-323^ | -1.03  (-1.06,-1.01) | <10^-323^ | -1.03  (-1.06,-0.99) | <10^-323^ | -1.03  (-1.07,-0.99) | <10^-323^ |
| *Sex (female)* | 0.01  (-0.03,0.05) | 0.68 | -0.10  (-0.16,-0.04) | 1.60E^-03^ | -0.14  (-0.21,-0.06) | 2.73E^-04^ | -0.19  (-0.27,-0.10) | 3.30E^-05^ |
| *per 10 cm in height* |  |  | -0.08  (-0.11,-0.05) | 5.68E^-06^ | -0.09  (-0.13,-0.05) | 2.70E^-06^ | -0.11  (-0.15,-0.06) | 4.76E^-06^ |
| *BMI* |  |  |  |  | -0.02  (-0.02,-0.01) | 4.92E^-08^ | -0.02  (-0.02,-0.01) | 2.18E^-06^ |
| *HbA1c mmol/mol* |  |  |  |  | -0.01  (-0.01,0.00) | 2.28E^-04^ | -0.01  (-0.01,0.00) | 0.02 |
| *per 10 mmHg in SBP* |  |  |  |  | 0.02  (0.00,0.03) | 0.02 | 0.02  (0.00,0.04) | 0.02 |
| *cholesterol mmol/L* |  |  |  |  | 0.01  (-0.02,0.03) | 0.57 | 0.00  (-0.03,0.03) | 0.86 |
| *triglycerides mmol/L* |  |  |  |  | 0.00  (-0.03,0.03) | 0.87 | 0.00  (-0.04,0.03) | 1.0 |

† - Coefficients as % change for log transformed target variables. * - Coefficients multiplied by 100. Model 1 - adjusted for age, sex, ethnicity, and UK Biobank centre. Model 2 - Model 1 adjustment, with adjustment for smoking, Townsend deprivation index and height. Model 3 – Model 2 adjustment, with adjustment for BMI, HbA1c, systolic BP, total cholesterol and triacylglycerols. Model 4 – Same as Model 3 but excluding persons with self-reported history of heart attack, stroke, hypertension or on medication for hypertension.

**eTable 10: Difference in retinal vasculometry characteristics per SD increase in log transformed reaction time (RS) and other covariates from multivariable regression models with different levels of adjustment.**

|  | **Model 1** | | **Model 2** | | **Model 3** | | **Model 4** | |
| --- | --- | --- | --- | --- | --- | --- | --- | --- |
|  | **Difference (95% CI)** | **p-value** | **Difference (95% CI)** | **p-value** | **Difference (95% CI)** | **p-value** | **Difference (95% CI)** | **p-value** |
| **Arteriolar width** |  |  |  |  |  |  |  |  |
| *per SD in RS* | 0.02  (-0.04,0.08) | 5.49E^-01^ | 0.04  (-0.02,0.10) | 0.23 | 0.02  (-0.05,0.09) | 0.60 | 0.03  (-0.05,0.11) | 0.45 |
| *per decade in age* | -0.87  (-0.95,-0.79) | 9.94E^-106^ | -0.65  (-0.73,-0.57) | 2.18E^-57^ | -0.10  (-0.19,0.00) | 0.04 | -0.07  (-0.17,0.04) | 0.23 |
| *Sex (female)* | -0.33  (-0.45,-0.21) | 5.06E^-08^ | 1.40  (1.22,1.57) | 5.31E^-57^ | 0.87  (0.68,1.06) | 1.06E^-18^ | 0.88  (0.66,1.11) | 1.82E^-14^ |
| *per 10 cm in height* |  |  | 1.29  (1.20,1.38) | 2.24E^-159^ | 1.19  (1.09,1.29) | 4.36E^-115^ | 1.25  (1.13,1.37) | 9.92E^-94^ |
| *BMI* |  |  |  |  | -0.02  (-0.04,-0.01) | 0.01 | -0.02  (-0.03,0.00) | 0.11 |
| *HbA1c mmol/mol* |  |  |  |  | 0.03  (0.02,0.05) | 5.57E^-10^ | 0.03  (0.02,0.05) | 2.11E^-05^ |
| *per 10 mmHg in SBP* |  |  |  |  | -0.85  (-0.89,-0.81) | <10^-323^ | -0.92  (-0.97,-0.87) | <10^-323^ |
| *cholesterol mmol/L* |  |  |  |  | -0.09  (-0.15,-0.02) | 0.01 | -0.05  (-0.12,0.02) | 0.18 |
| *triglycerides mmol/L* |  |  |  |  | 0.08  (0.00,0.15) | 0.04 | 0.07  (-0.02,0.16) | 0.11 |
| **Venular width** |  |  |  |  |  |  |  |  |
| *per SD in RS* | -0.03  (-0.13,0.08) | 0.62 | -0.04  (-0.15,0.06) | 0.44 | -0.06  (-0.17,0.06) | 0.34 | -0.01  (-0.15,0.12) | 0.83 |
| *per decade in age* | 0.57  (0.44,0.70) | 1.07E^-17^ | 0.71  (0.58,0.84) | 2.22E^-25^ | 0.80  (0.64,0.96) | 2.42E^-23^ | 0.78  (0.60,0.97) | 1.26E^-16^ |
| *Sex (female)* | -0.81  (-1.01,-0.61) | 9.60E^-16^ | 0.10  (-0.19,0.39) | 0.49 | 0.19  (-0.14,0.52) | 0.26 | 0.36  (-0.03,0.74) | 0.07 |
| *per 10 cm in height* |  |  | 0.63  (0.47,0.79) | 3.22E^-15^ | 0.66  (0.48,0.83) | 1.31E^-13^ | 0.69  (0.49,0.90) | 4.18E^-11^ |
| *BMI* |  |  |  |  | 0.10  (0.08,0.13) | 1.15E^-15^ | 0.11  (0.08,0.14) | 1.48E^-11^ |
| *HbA1c mmol/mol* |  |  |  |  | 0.03  (0.01,0.05) | 1.13E^-03^ | 0.04  (0.01,0.07) | 3.33E^-03^ |
| *per 10 mmHg in SBP* |  |  |  |  | -0.29  (-0.36,-0.23) | 1.90E^-18^ | -0.29  (-0.37,-0.21) | 4.80E^-12^ |
| *cholesterol mmol/L* |  |  |  |  | 0.02  (-0.08,0.12) | 0.73 | -0.04  (-0.17,0.08) | 0.49 |
| *triglycerides mmol/L* |  |  |  |  | 0.35  (0.22,0.48) | 7.78E^-08^ | 0.41  (0.25,0.56) | 2.93E^-07^ |
| **Arteriolar tortuosity**† |  |  |  |  |  |  |  |  |
| *per SD in RS* | 0.06  (-0.35,0.47) | 0.76 | 0.04  (-0.37,0.45) | 0.85 | 0.04  (-0.42,0.49) | 0.88 | 0.10  (-0.44,0.64) | 0.72 |
| *per decade in age* | 2.07  (1.55,2.59) | 3.06E^-15^ | 2.12  (1.59,2.66) | 5.57E^-15^ | 0.96  (0.33,1.59) | 2.79E^-03^ | 0.61  (-0.13,1.35) | 0.10 |
| *Sex (female)* | 3.85  (3.05,4.66) | 1.55E^-21^ | 3.58  (2.41,4.75) | 1.07E^-09^ | 4.69  (3.33,6.06) | 5.65E^-12^ | 4.85  (3.26,6.47) | 1.37E^-09^ |
| *per 10 cm in height* |  |  | -0.24  (-0.85,0.38) | 0.45 | -0.11  (-0.80,0.58) | 0.76 | -0.18  (-0.99,0.63) | 0.66 |
| *BMI* |  |  |  |  | -0.15  (-0.25,-0.05) | 3.11E^-03^ | -0.31  (-0.44,-0.18) | 2.11E^-06^ |
| *HbA1c mmol/mol* |  |  |  |  | 0.12  (0.05,0.20) | 1.09E^-03^ | 0.09  (-0.02,0.19) | 0.10 |
| *per 10 mmHg in SBP* |  |  |  |  | 1.32  (1.05,1.58) | 7.42E^-23^ | 1.18  (0.85,1.51) | 1.62E^-12^ |
| *cholesterol mmol/L* |  |  |  |  | -0.02  (-0.43,0.39) | 0.91 | 0.26  (-0.25,0.76) | 0.32 |
| *triglycerides mmol/L* |  |  |  |  | 0.62  (0.11,1.13) | 0.02 | 0.72  (0.11,1.34) | 0.02 |
| **Venular tortuosity**† |  |  |  |  |  |  |  |  |
| *per SD in RS* | 0.24  (-0.01,0.49) † | 0.05 | 0.20  (-0.05,0.45) † | 0.12 | 0.13  (-0.14,0.41) † | 0.34 | 0.11  (-0.20,0.43) † | 0.48 |
| *per decade in age* | 2.39  (2.08,2.71) † | 7.80E^-52^ | 2.47  (2.15,2.79) † | 9.44E^-52^ | 1.99  (1.61,2.37) † | 4.53E^-25^ | 1.82  (1.38,2.26) † | 3.06E^-16^ |
| *Sex (female)* | 1.31  (0.83,1.78) † | 5.17E^-08^ | 1.10  (0.42,1.79) † | 1.52E^-03^ | 1.94  (1.15,2.73) † | 1.28E^-06^ | 1.71  (0.80,2.64) † | 2.37E^-04^ |
| *per 10 cm in height* |  |  | -0.19  (-0.56,0.18) † | 0.32 | 0.09  (-0.32,0.50) † | 0.68 | 0.13  (-0.35,0.61) † | 0.60 |
| *BMI* |  |  |  |  | 0.42  (0.36,0.48) † | 5.97E^-42^ | 0.29  (0.21,0.36) † | 1.39E^-13^ |
| *HbA1c mmol/mol* |  |  |  |  | 0.13  (0.09,0.18) † | 4.00E^-09^ | 0.10  (0.04,0.16) † | 1.63E-03 |
| *per 10 mmHg in SBP* |  |  |  |  | 0.48  (0.33,0.64) † | 1.12E^-09^ | 0.42  (0.22,0.61) † | 2.13E^-05^ |
| *cholesterol mmol/L* |  |  |  |  | -0.44  (-0.68,-0.19) † | 4.15E^-04^ | -0.11  (-0.41,0.19) † | 0.46 |
| *triglycerides mmol/L* |  |  |  |  | -0.46  (-0.76,-0.16) † | 2.84E^-03^ | -0.54  (-0.90,-0.18) † | 3.16E^-03^ |
| **Arteriolar area** |  |  |  |  |  |  |  |  |
| *per SD in RS* | -0.80  (-1.42,-0.18)* | 0.01 | -0.72  (-1.34,-0.09)* | 0.02 | -0.68  (-1.37,0.00)* | 0.05 | -0.62  (-1.45,0.20)* | 0.14 |
| *per decade in age* | -0.36  (-0.37,-0.35) | <10^-323^ | -0.35  (-0.36,-0.34) | <10^-323^ | -0.31  (-0.32,-0.30) | <10^-323^ | -0.32  (-0.33,-0.31) | <10^-323^ |
| *Sex (female)* | -0.04  (-1.23,1.14) * | 0.94 | 0.08  (0.06,0.09) | 2.48E^-18^ | 0.02  (0.01,0.04) | 0.01 | 0.02  (-0.01,0.04) | 0.15 |
| *per 10 cm in height* |  |  | 0.06  (0.05,0.07) | 4.22E^-33^ | 0.05  (0.04,0.06) | 8.03E^-19^ | 0.05  (0.04,0.06) | 5.68E^-14^ |
| *BMI* |  |  |  |  | -0.01  (-0.01,-0.01) | 1.45E^-33^ | -0.01  (-0.01,-0.01) | 2.47E^-19^ |
| *HbA1c mmol/mol* |  |  |  |  | 0.01  (-0.10,0.12)* | 0.90 | 0.08  (-0.08,0.24)* | 0.32 |
| *per 10 mmHg in SBP* |  |  |  |  | -0.06  (-0.06,-0.05) | 1.34E^-173^ | -0.06  (-0.06,-0.05) | 4.07E^-117^ |
| *cholesterol mmol/L* |  |  |  |  | 0.28  (-0.33,0.90)* | 0.36 | -0.08  (-0.85,0.69)* | 0.83 |
| *triglycerides mmol/L* |  |  |  |  | -0.36  (-1.11,0.40)* | 0.36 | 0.25  (-0.68,1.19)* | 0.59 |
| **Venular area** |  |  |  |  |  |  |  |  |
| *per SD in RS* | -0.01  (-0.01,0.00) | 0.03 | -0.01  (-0.01,0.00) | 0.14 | 0.00  (-0.01,0.00) | 0.34 | 0.00(-0.01,0.01) | 0.91 |
| *per decade in age* | -0.08  (-0.08,-0.07) | 7.54E^-66^ | -0.06  (-0.07,-0.05) | 3.27E^-41^ | -0.05  (-0.06,-0.04) | 2.98E^-19^ | -0.05(-0.06,-0.04) | 2.92E^-16^ |
| *Sex (female)* | -0.21  (-0.23,-0.20) | 6.65E^-221^ | -0.08  (-0.10,-0.06) | 2.48E^-16^ | -0.08  (-0.10,-0.06) | 3.08E^-12^ | -0.07(-0.10,-0.05) | 8.51E^-08^ |
| *per 10 cm in height* |  |  | 0.10  (0.09,0.11) | 1.51E^-78^ | 0.10  (0.09,0.11) | 1.85E^-66^ | 0.10(0.09,0.12) | 4.81E^-48^ |
| *BMI* |  |  |  |  | 0.01  (0.01,0.01) | 7.11E^-25^ | 0.01(0.01,0.01) | 1.07E^-12^ |
| *HbA1c mmol/mol* |  |  |  |  | 0.08  (-0.05,0.20)* | 0.24 | 0.06(-0.11,0.24)* | 0.47 |
| *per 10 mmHg in SBP* |  |  |  |  | -0.02  (-0.03,-0.02) | 1.07E^-27^ | -0.02(-0.03,-0.02) | 4.75E^-17^ |
| *cholesterol mmol/L* |  |  |  |  | 0.01  (0.00,0.02) | 8.35E^-04^ | 0.01  (0.00,0.02) | 5.28E^-03^ |
| *triglycerides mmol/L* |  |  |  |  | 0.01  (0.00,0.02) | 0.03 | 0.01  (0.00,0.02) | 0.03 |
| **1/ (Arteriolar SD(µm))*** |  |  |  |  |  |  |  |  |
| *per SD in RS* | 0.01  (-0.01,0.03) | 0.25 | 0.01  (-0.01,0.03) | 0.26 | 0.01  (-0.01,0.03) | 0.34 | 0.01  (-0.01,0.04) | 0.30 |
| *per decade in age* | -0.92  (-0.94,-0.89) | <10^-323^ | -0.92  (-0.95,-0.90) | <10^-323^ | -0.84  (-0.87,-0.81) | <10^-323^ | -0.86  (-0.90,-0.82) | <10^-323^ |
| *Sex (female)* | 0.09  (0.05,0.12) | 8.09E^-06^ | 0.03  (-0.03,0.08) | 0.31 | -0.06  (-0.12,0.01) | 0.07 | -0.08  (-0.16,-0.01) | 0.03 |
| *per 10 cm in height* |  |  | -0.04  (-0.07,-0.01) | 0.01 | -0.06  (-0.09,-0.03) | 4.96E^-04^ | -0.05  (-0.09,-0.01) | 0.01 |
| *BMI* |  |  |  |  | -0.01  (-0.01,0.00) | 1.31E^-03^ | -0.01  (-0.02,-0.01) | 1.94E^-04^ |
| *HbA1c mmol/mol* |  |  |  |  | 0.00  (-0.01,0.00) | 0.26 | 0.00  (-0.01,0.00) | 0.20 |
| *per 10 mmHg in SBP* |  |  |  |  | -0.11  (-0.12,-0.09) | 1.91E^-61^ | -0.11  (-0.13,-0.09) | 4.01E^-41^ |
| *cholesterol mmol/L* |  |  |  |  | -0.02  (-0.04,0.00) | 0.07 | 0.00  (-0.03,0.02) | 0.82 |
| *triglycerides mmol/L* |  |  |  |  | 0.00  (-0.03,0.02) | 0.90 | -0.01  (-0.04,0.02) | 0.59 |
| **1/ (Venular SD(µm))*** |  |  |  |  |  |  |  |  |
| *per SD in RS* | 0.00  (-0.02,0.02) | 0.96 | 0.00  (-0.02,0.02) | 0.94 | 0.00  (-0.03,0.02) | 0.92 | 0.00  (-0.03,0.03) | 0.88 |
| *per decade in age* | -1.01  (-1.04,-0.98) | <10^-323^ | -1.03  (-1.06,-1.00) | <10^-323^ | -1.02  (-1.06,-0.99) | <10^-323^ | -1.02  (-1.06,-0.98) | <10^-323^ |
| *Sex (female)* | 0.01  (-0.03,0.05) | 0.67 | -0.10  (-0.17,-0.04) | 1.37E^-03^ | -0.14  (-0.21,-0.06) | 2.46E^-04^ | -0.19  (-0.27,-0.10) | 3.01E^-05^ |
| *per 10 cm in height* |  |  | -0.08  (-0.12,-0.05) | 3.86E^-06^ | -0.09  (-0.13,-0.06) | 1.95E^-06^ | -0.11  (-0.16,-0.06) | 3.59E^-06^ |
| *BMI* |  |  |  |  | -0.02  (-0.02,-0.01) | 3.51E^-08^ | -0.02  (-0.03,-0.01) | 1.80E^-06^ |
| *HbA1c mmol/mol* |  |  |  |  | -0.01  (-0.01,0.00) | 2.34E^-04^ | -0.01  (-0.01,0.00) | 0.02 |
| *per 10 mmHg in SBP* |  |  |  |  | 0.02  (0.00,0.03) | 0.02 | 0.02  (0.00,0.04) | 0.02 |
| *cholesterol mmol/L* |  |  |  |  | 0.01  (-0.02,0.03) | 0.58 | 0.00  (-0.03,0.03) | 0.84 |
| *triglycerides mmol/L* |  |  |  |  | 0.00  (-0.03,0.03) | 0.87 | 0.00  (-0.04,0.03) | 0.95 |

† - Coefficients as % change for log transformed target variables. * - Coefficients multiplied by 100. Model 1 - adjusted for age, sex, ethnicity, and UK Biobank centre. Model 2 - Model 1 adjustment, with adjustment for smoking, Townsend deprivation index and height. Model 3 – Model 2 adjustment, with adjustment for BMI, HbA1c, systolic BP, total cholesterol and triacylglycerols. Model 4 – Same as Model 3 but excluding persons with self-reported history of heart attack, stroke, hypertension or on medication for hypertension.

**eFigure 1: Scree plot of eigenvalues generated from the 4 UK Biobank cognitive tests (only the first principal component (G4) with an eigen value greater than 1 was used.**

**
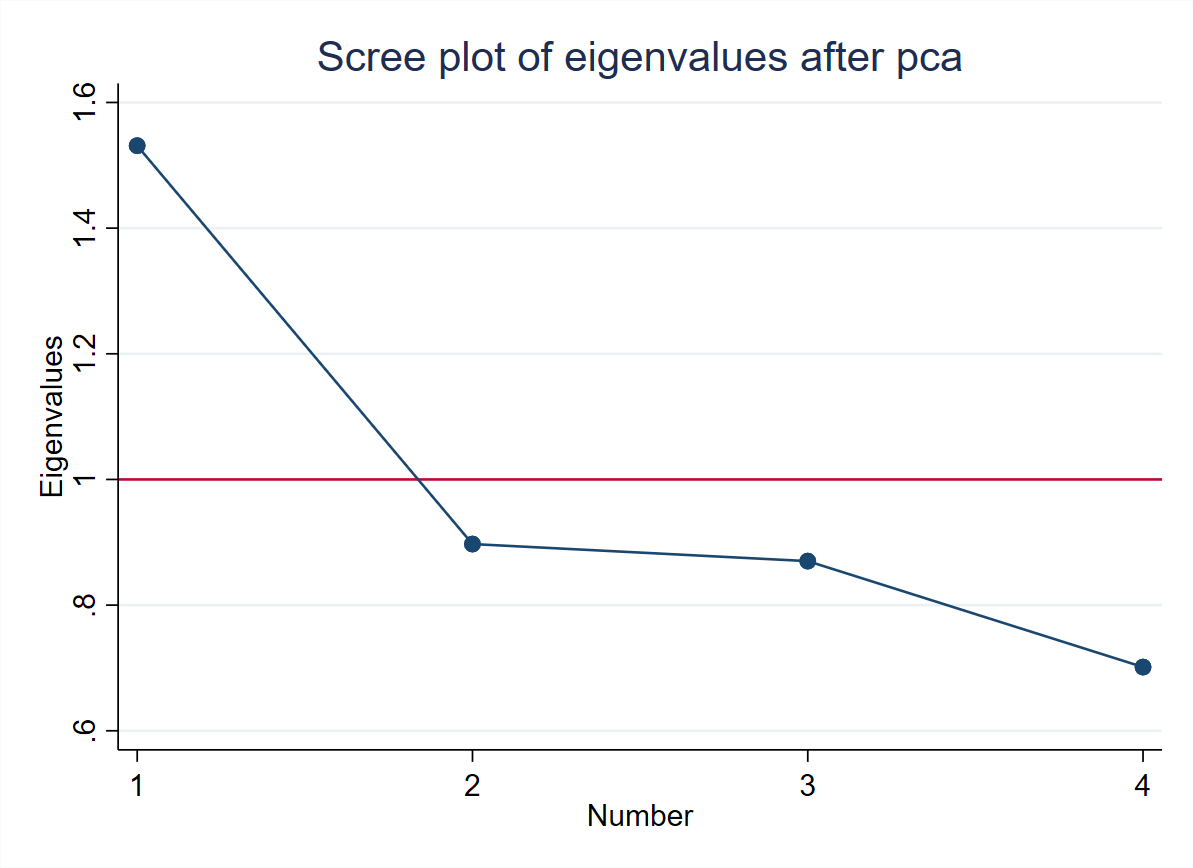
**

**eFigure 2: Histogram of PCA score (G4) for the first unrotated principal component, representing the overall cognitive score.**

**
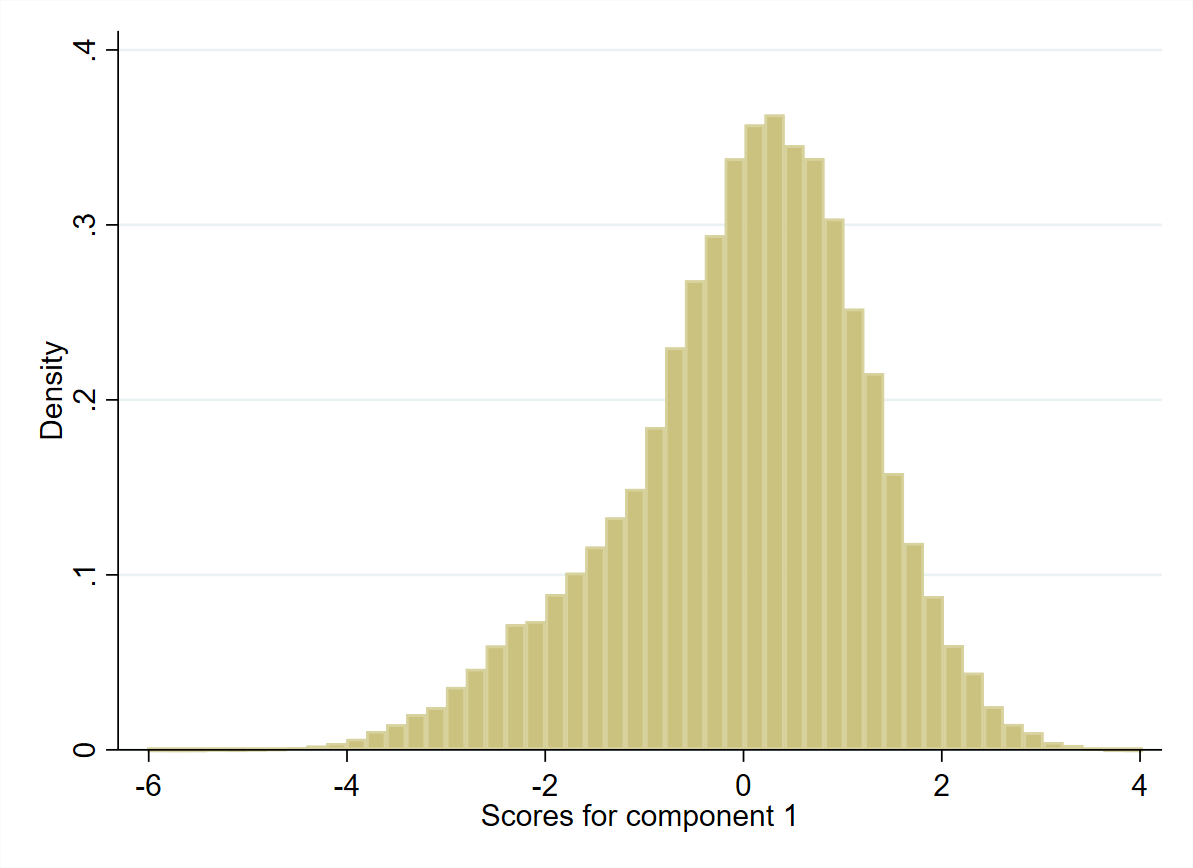
**

***eFigure 3: QUARTZ output for retinal images. The RV measures corresponding to the numbered segments in retinal images are then used to compute average RV measures weighted by segment length.***

***
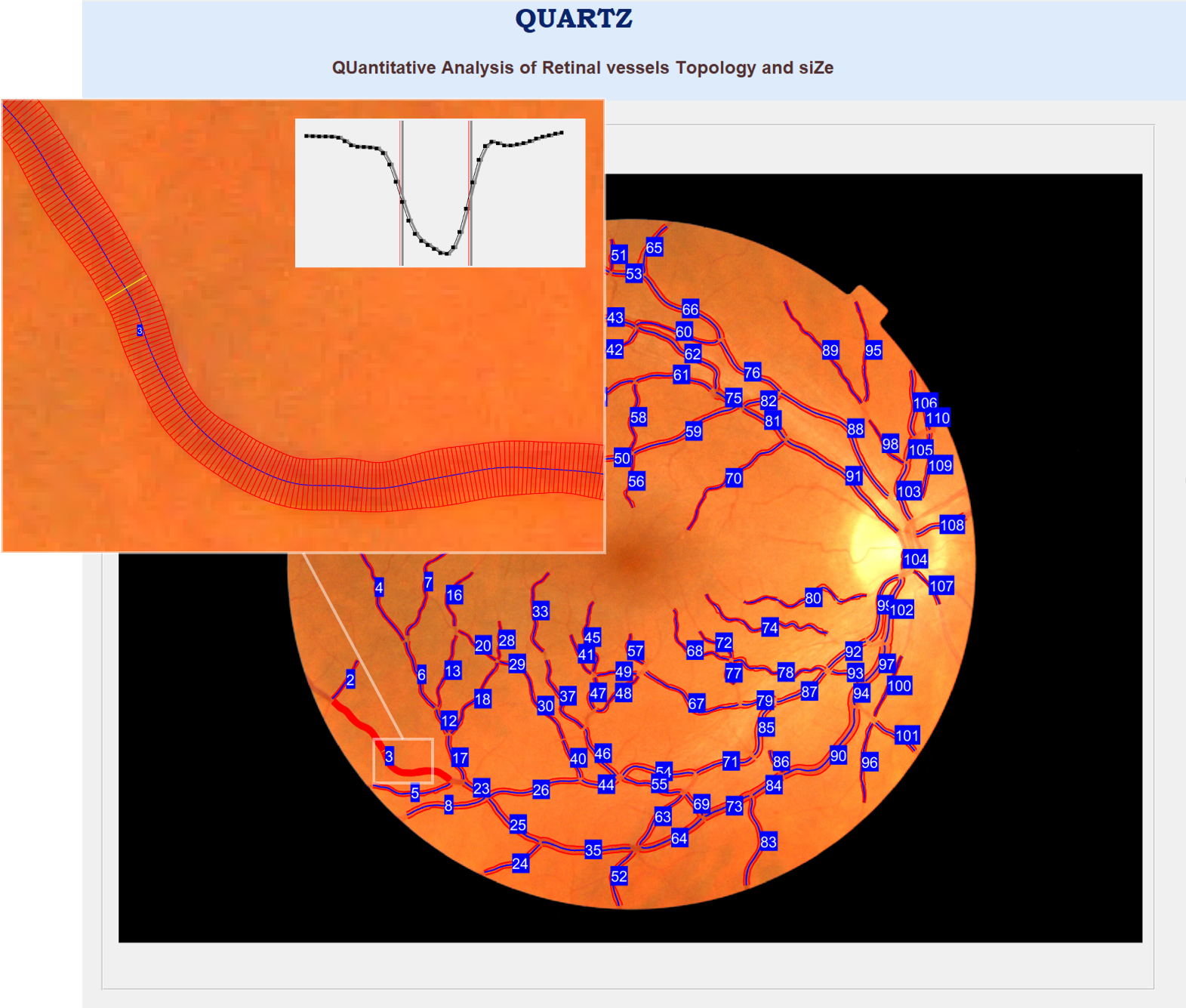
***

**eFigure 4: Flow diagram of UK Biobank participants used in the analyses**


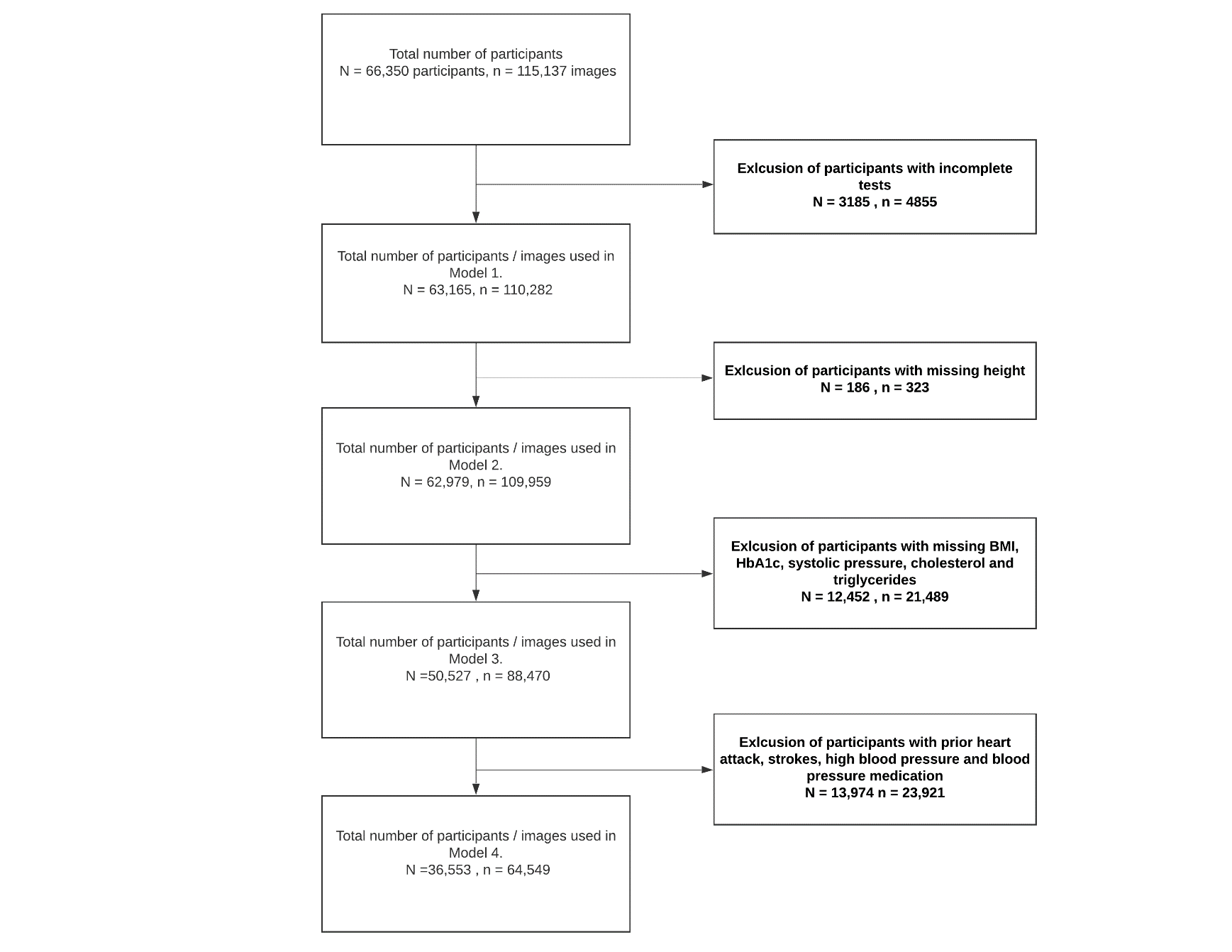


**eFigure 5: Adjusted mean retinal vasculometry characteristics by deciles / quintiles of G4 for each ethnic group.**


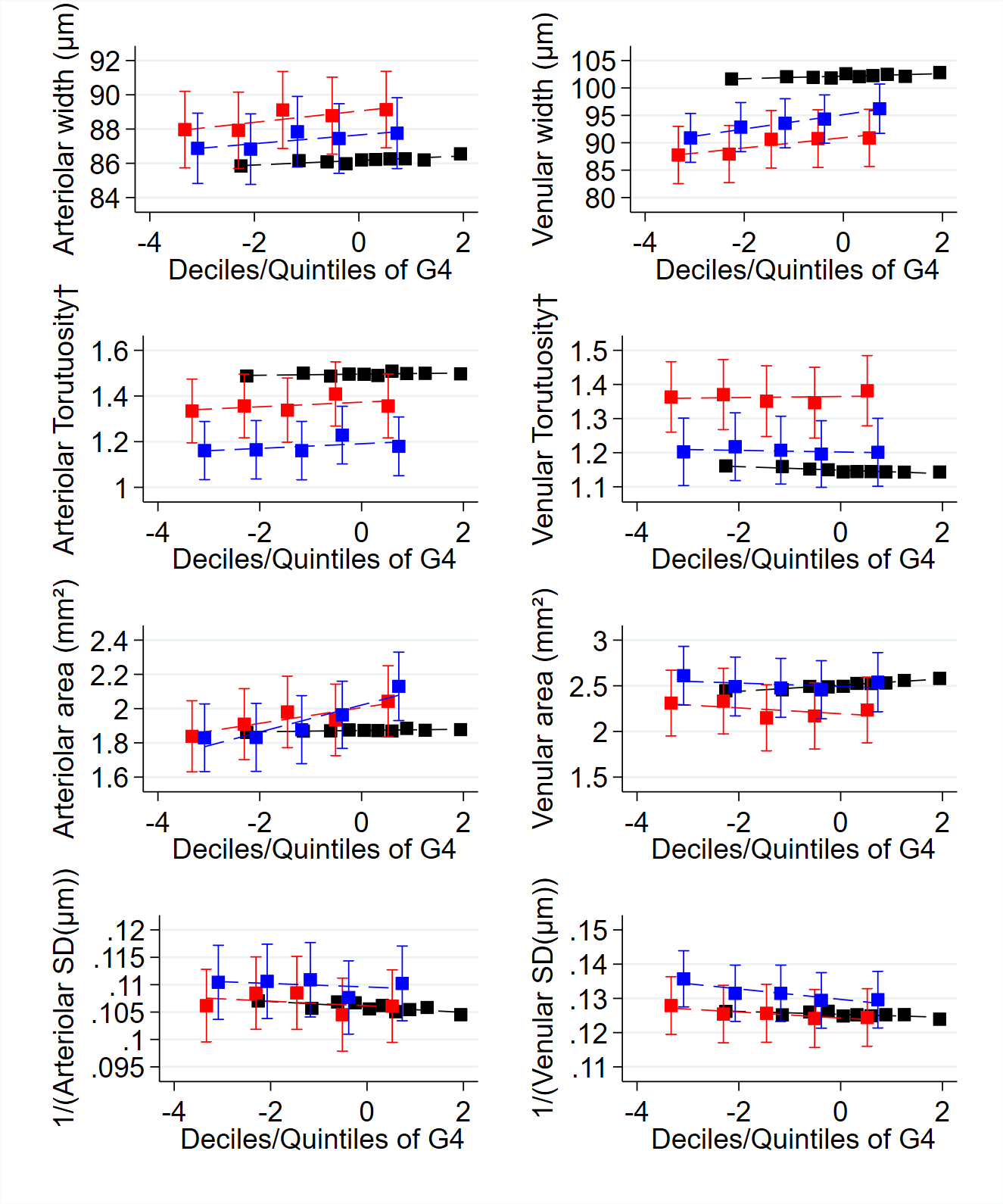


***Adjusted means (solid square symbols), 95% CIs (solid vertical error bars) and regression line (dotted line) of retinal vasculometry by deciles (White group) /quintiles (Black group, Asians) of G4, are from multilevel models stratified by ethnicity adjusted for age, gender, UK Biobank centre as fixed effects, allowing for repeated retinal vessel measures within each person (N participant = 63165, n images = 110282). Black, red, and blue represent White, Black, and Asian ethnic groups, respectively. † Natural log transformed values.***

**eFigure 6: Adjusted mean retinal vasculometry characteristics by sextiles of fluid intelligence score.**

**
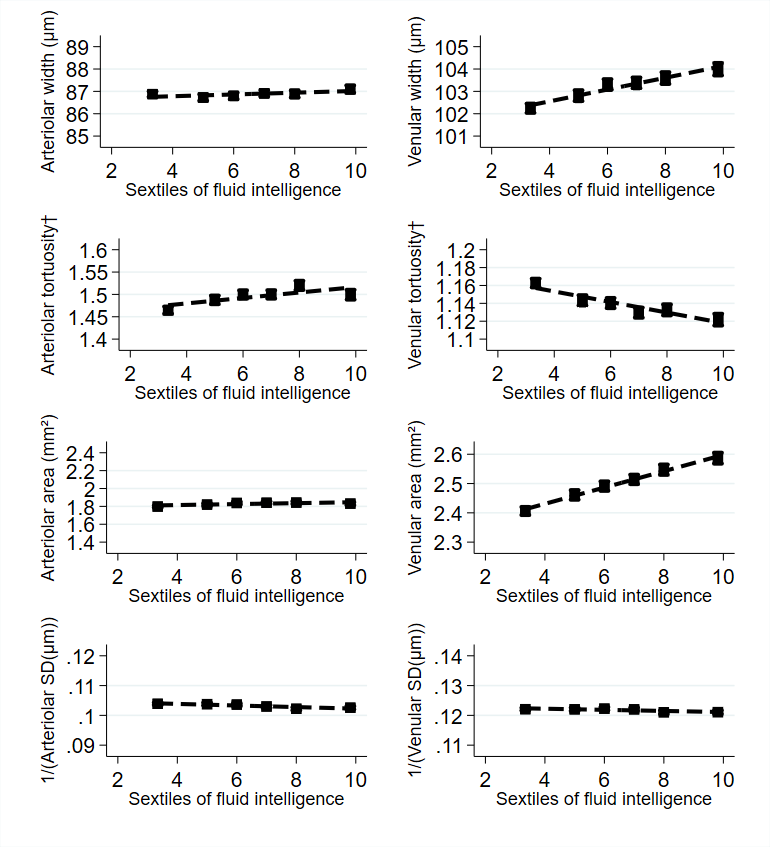
**

***Adjusted means (solid square symbols), 95% CIs (solid vertical error bars) and regression line (dashed line) for retinal vasculometry, by deciles of log transformed fluid intelligence score, from multilevel models adjusted for age, gender, and as fixed effects, allowing for repeated retinal vasculometry measures within each person (N participant = 63165, n images = 110282). † Natural log transformed values.***

**eFigure 7: Adjusted mean retinal vasculometry characteristics by deciles of pairs matching score.**

**
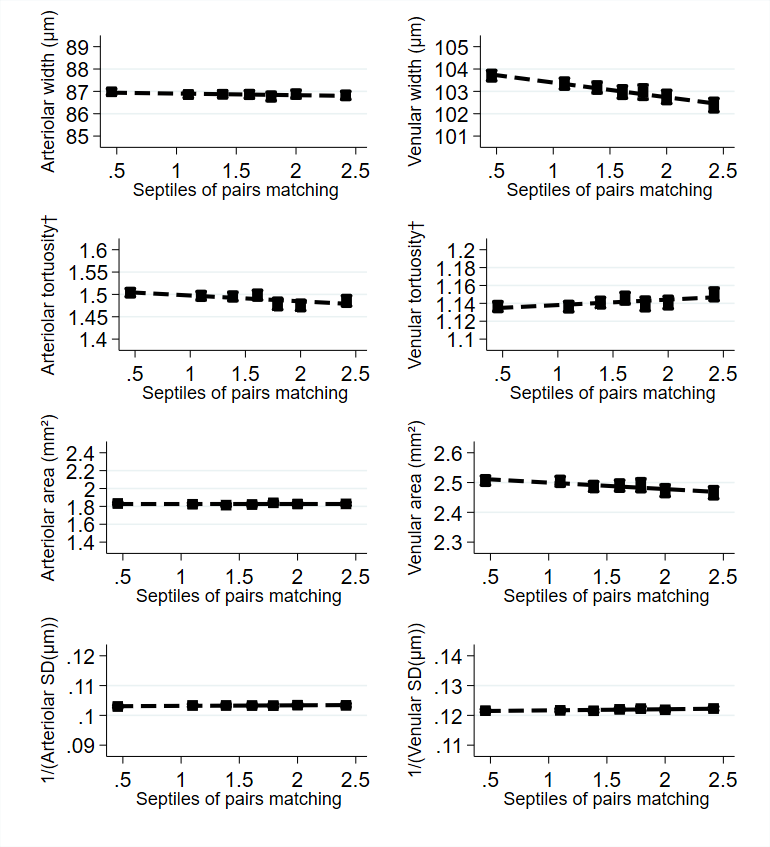
**

***Adjusted means (solid square symbols), 95% CIs (solid vertical error bars) and regression line (dashed line) for retinal vasculometry, by septiles of log transformed pairs matching scores, from multilevel models adjusted for age, gender, and as fixed effects, allowing for repeated retinal vasculometry measures within each person (N participant = 63165, n images = 110282). † Natural log transformed values.***

**eFigure 8: Adjusted mean retinal vasculometry characteristics by deciles of reaction time.**

**
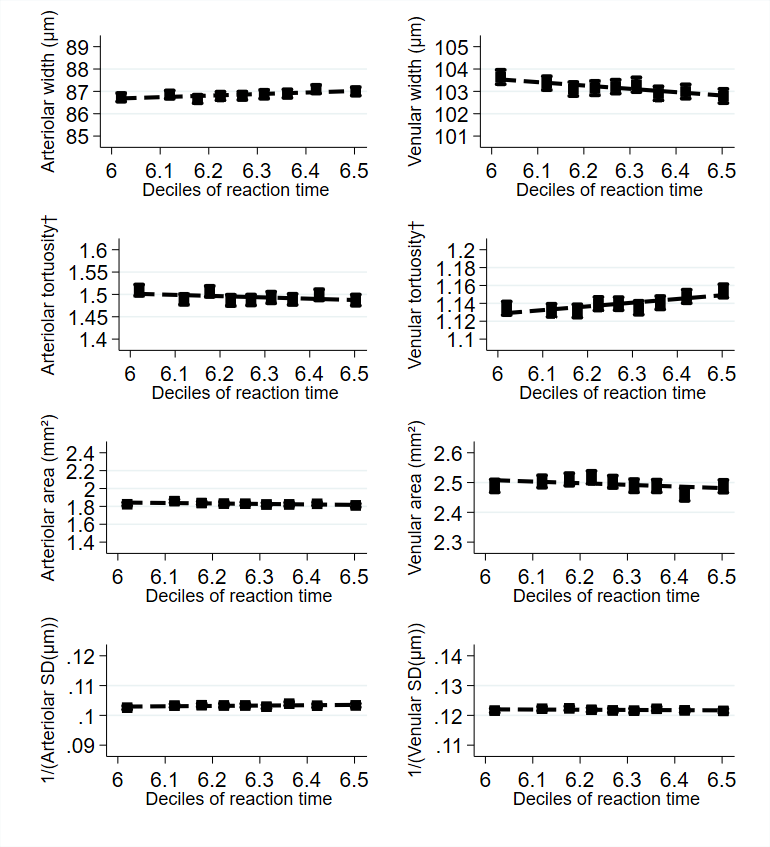
**

***Adjusted means (solid square symbols), 95% CIs (solid vertical error bars) and regression line (dashed line) for retinal vasculometry, by deciles of log transformed reaction time, from multilevel models adjusted for age, gender, allowing for repeated retinal vasculometry measures within each person (N participant = 63165, n images = 110282). † Natural log transformed values.***

**Supplemental text from the Discussion with e-references**

The concept that the eye provides a window on neurodegenerative processes within the central nervous system is well established. Degeneration of the optic nerve is a well-recognized feature of AD,[12] and a growing body of evidence (including work carried in UK Biobank) has shown that total and segmented thicknesses of the neuroretina gives insights into both cognitive function and volumetric characteristics of the brain relevant to cognition.[8,10] People suffering AD have a thinner retinal nerve fibre layer (RNFL) and retinal ganglion cell layer (GCL) than those without AD of a similar age, and OCT measurement of RNFL and GCL may be useful in discriminating between AD and healthy individuals.[48,49] These findings provide a potential biological basis for the RV associations observed in that these neuronal changes may be as a result of or a consequence of reduced axonal activity and function.[13] Moreover, the concept that the retina may allow early detection of systemic and non-ocular disease (in addition to ocular neurodegenerative disorders, such as glaucoma) is well-established for other vascular related diseases.[50, e51-e53] We believe the combination of our unique method of RV assessment (as a marker of retinal vasculopathy and microvascular dysfunction) along with the strong graded associations observed, has a strong basis as a predictor of cognitive decline and subsequent neurodegenerative disease. Prediction approaches may need to include different RV characteristics, and be nuanced to account for different vasculometry associations, e.g., for venular width and venular area where there was evidence of effect modification in association by age. We have previously demonstrated such prediction approaches for circulatory mortality, CHD and stroke in UK Biobank, which perform as well as established risk scores.[27] For neurodegenerative disease, the additive value of RNFL along with retinal sub-layer measures, may be incremental.[e54]

This study has strengths and weaknesses. UK Biobank offers one of the largest and most widely phenotyped retinal imaging data-sets in the world.[36] Even though macular centred retinal images were captured by non-experts, inclusion rates were high, limiting potential selection biases.[27] We also distinguished between arterioles and venules, which could show different RV-cognitive associations. While UK Biobank includes those largely of white ethnic origin, numbers of participants of non-white ancestry were still high, allowing the consistency of RV-cognitive associations across ethnic groups to be examined. However, replication of these findings in other large diverse data-sources would still be worthwhile. UK Biobank is a ‘healthy’ cohort compared with other similarly aged nationally representative cohorts,[e55] and appreciable numbers with AD are yet to evolve, although, the age of participants remains optimal to examine those most likely to experience age related cognitive decline, a well-established precursor of neurodegenerative disease.[e56,e57] However, those included with useable retinal images were younger and healthier, which might not reflect the full spectrum of cognitive status. It is noteworthy that the cognitive tests used to measure cognitive decline were specifically developed for UK Biobank with unknown validity and test-retest reliability.[34,35] However, the test performance of these cognitive tests,[35] and their association with subsequent neurodegenerative diseases (particularly AD), have since been shown.[e58]

The ease, speed and precision of AI derived vascular metrics generated from retinal imaging, and the strong definitive associations these show with cognitive status, may offer a biomarker to more accurately discriminate between those who subsequently develop neurodegenerative outcomes from those who do not. This biomarker could be enhanced by ongoing technological improvements in image capture, coupled with the additive value OCT RNFL measures, in addition to deeper vascular OCT-A assessment, which in time could become more routinely available.[18] OCT RNFL thicknesses are available in UK Biobank but we are yet to examine their associations with these RV measures. Examining the time course of structural changes in the retina associated with cognitive decline and how these relate to RV changes, will provide further insight into the mechanism of disease. However, for now, the relative to the high cost of hospital- or clinic-based brain scanning or blood testing, CFP are low cost to acquire, non-invasive, rapid and scalable given availability within existing opticians and eye clinic healthcare pathways, maximizing population reach, providing the potential to screen for cognitive decline and intervene early to avert / delay age-related neurodegenerative outcomes.[48,49]

e51. Ikram MK, Ong YT, Cheung CY, Wong TY. Retinal vascular caliber measurements: clinical significance, current knowledge and future perspectives. *Ophthalmologica* 2013; **229**(3): 125-36.

e52. Cheung CY, Ikram MK, Klein R, Wong TY. The clinical implications of recent studies on the structure and function of the retinal microvasculature in diabetes. *Diabetologia* 2015; **58**(5): 871-85.

e53. Seidelmann SB, Claggett B, Bravo PE, Gupta A, Farhad H, Klein BE, Klein R, Di Carli M, Solomon SD. Retinal Vessel Calibers in Predicting Long-Term Cardiovascular Outcomes: The Atherosclerosis Risk in Communities Study. *Circulation* 2016; **134**(18): 1328-38.

e54. Alber J, Goldfarb D, Thompson LI, Arthur E, Hernandez K, Cheng D, DeBuc DC, Cordeiro F, Provetti-Cunha L, den Haan J, Van Stavern GP, Salloway SP, Sinoff S, Snyder PJ. Developing retinal biomarkers for the earliest stages of Alzheimer's disease: What we know, what we don't, and how to move forward. *Alzheimers Dement* 2020; **16**(1): 229-43.

e55. Owen CG, Kapetanakis VV, Rudnicka AR, Wathern AK, Lennon L, Papacosta O, Cook DG, Wannamethee SG, Whincup PH. Body mass index in early and middle adult life: prospective associations with myocardial infarction, stroke and diabetes over a 30-year period: the British Regional Heart Study. *BMJ Open* 2015; **5**(9): e008105.

e56. Singh-Manoux A, Kivimaki M, Glymour MM, Elbaz A, Berr C, Ebmeier KP, Ferrie JE, Dugravot A. Timing of onset of cognitive decline: results from Whitehall II prospective cohort study. *BMJ* 2012; **344**: d7622.

e57. Jack CR, Jr., Bennett DA, Blennow K, Carrillo MC, Dunn B, Haeberlein SB, Holtzman DM, Jagust W, Jessen F, Karlawish J, Liu E, Molinuevo JL, Montine T, Phelps C, Rankin KP, Rowe CC, Scheltens P, Siemers E, Snyder HM, Sperling R, Contributors. NIA-AA Research Framework: Toward a biological definition of Alzheimer's disease. *Alzheimers Dement* 2018; **14**(4): 535-62.

e58. Swaddiwudhipong N, Whiteside DJ, Hezemans FH, Street D, Rowe JB, Rittman T. Pre-diagnostic cognitive and functional impairment in multiple sporadic neurodegenerative diseases. *Alzheimers Dement* 2022.
